# Conserved patterns of integrated developmental plasticity in a group of polyphenic tropical butterflies

**DOI:** 10.1186/s12862-017-0907-1

**Published:** 2017-02-27

**Authors:** Erik van Bergen, Dave Osbaldeston, Ullasa Kodandaramaiah, Oskar Brattström, Kwaku Aduse-Poku, Paul M. Brakefield

**Affiliations:** 1 Department of Zoology, University of Cambridge, Downing Street, Cambridge, CB2 3EJ UK; 2 School of Biology, Indian Institute of Science Education and Research Thiruvananthapuram, CET campus, Trivandrum, 695016 India; 3 Present Address: Instituto Gulbenkian de Ciência, Rua da Quinta Grande 6, P-2780 Oeiras, Portugal; 4 Present Address: Department of Biology, City College of New York, City University of New York, Convent Avenue at 138th Street, New York, NY 10031 USA

**Keywords:** Mycalesine butterflies, Developmental plasticity, Seasonal polyphenism, Reaction norm, Life-history evolution, *Bicyclus anynana*

## Abstract

**Background:**

Developmental plasticity is thought to have profound macro-evolutionary effects, for example, by increasing the probability of establishment in new environments and subsequent divergence into independently evolving lineages. In contrast to plasticity optimized for individual traits, phenotypic integration, which enables a concerted response of plastic traits to environmental variability, may affect the rate of local adaptation by constraining independent responses of traits to selection. Using a comparative framework, this study explores the evolution of reaction norms for a variety of life history and morphological traits across five related species of mycalesine butterflies from the Old World tropics.

**Results:**

Our data indicate that an integrated response of a suite of key traits is shared amongst these species. Interestingly, the traits that make up the functional suite are all known to be regulated by ecdysteroid signalling in *Bicyclus anynana*, one of the species included in this study, suggesting the same underlying hormonal regulator may be conserved within this group of polyphenic butterflies. We also detect developmental thresholds for the expression of alternative morphs.

**Conclusions:**

The phenotypic plasticity of a broad suite of morphological and life history traits is integrated and shared among species from three geographically independent lineages of mycalesine butterflies, despite considerable periods of independent evolution and exposure to disparate environments. At the same time, we have detected examples of evolutionary change where independent traits show different patterns of reaction norms. We argue that the expression of more robust phenotypes may occur by shifting developmental thresholds beyond the boundaries of the typical environmental variation.

**Electronic supplementary material:**

The online version of this article (doi:10.1186/s12862-017-0907-1) contains supplementary material, which is available to authorized users.

## Background

Developmental plasticity, the capacity of a single genotype to generate a range of phenotypes through environmental regulation of development [1], is ubiquitous among plants and animals [2, 3]. Whether developmental plasticity constrains or facilitates adaptive evolution and macro-evolutionary diversification has been the topic of recent debate [4–7]. Adaptive developmental plasticity, wherein an inductive environmental cue triggers the development of a phenotype better suited to a forthcoming environment, allows individuals to cope with environmental heterogeneity across space or time [8, 9]. By altering the distribution of phenotypes in the same direction as that favoured by directional selection, non-heritable environmentally induced variation has been expected to constrain or slow the rate of adaptive evolution [10]. However, at a macro-evolutionary scale, adaptive plasticity is thought to facilitate the process of local adaptation and diversification by enabling populations to successfully invade novel environments and subsequently diverge into independently evolving lineages [11–13]. Empirical support for this theory is provided by a number of examples from plants and animals [14–18]. For instance, the repeated evolution of freshwater ecotypes in the three-spined stickleback (*Gasterosteus aculeatus*) radiation appears to have been driven by specific patterns of ancestral phenotypic plasticity in the founding oceanic population [19].

Adaptive plastic responses of key traits are often integrated into functional suites to enable a concerted response to external cues and corresponding internal signals [20]. For example, environmentally induced diapause in insects, which involves synchronized responses in behaviour, physiology, morphology, and life history traits, is mediated by common physiological-endocrine processes [21]. The integration and coordination of responses typically results in strong genetic and phenotypic correlations between plastic traits which may affect the rate of adaptation to novel environments by constraining the independent response of plastic traits to selection [22–24]. Comparative investigations of developmental plasticity, within a phylogenetic and ecological framework, have been used to infer the extent to which local adaptation is constrained by correlations between traits and to shed light on the ways by which plasticity may contribute to diversification [13]. Many of these studies have compared the shapes and slopes of reaction norms across environments in closely related species or ecotypes subject to different selection pressures [25–28]. The results of these studies show that synchronized phenotypic responses of plastic traits can become uncoupled during periods of independent evolution to facilitate expansions into novel environments.

The number of in-depth comparative analyses of developmental plasticity in insects is remarkably low, especially considering the significant contribution insect model-systems have made to our understanding of the phenomenon (see Beldade et al. [29]). A group of insects that is particularly interesting in this context is the butterfly subtribe Mycalesina (Nymphalidae: Satyrinae: Satyrini). Mycalesine butterflies have radiated dramatically in Africa (including Madagascar) and Asia yielding over 300 extant species that inhabit a range of tropical and sub-tropical habitats [30]. They began to diversify rapidly in the mid-Miocene when much of the trans-continental forests were replaced by a mosaic of more seasonal open woodland and savannah habitats [31, 32]. Many extant mycalesine species exhibit a striking mode of seasonal polyphenism. The phenomenon is particularly well studied in the African species *Bicyclus anynana* (Butler, 1879), which has become an important model organism for ecological, evolutionary and developmental biology [33–35]. Seasonal variation in ambient temperature and rainfall are positively correlated across the entire distribution of *B. anynana* in East Africa. A rise in temperature in open woodland habitats predicts the onset of the rainy season and an increase in host plant density. After this period of increased rainfall the temperature drops significantly and the environment gradually dries out [36]. The predictable relationship between these abiotic factors is thought to have selected for a predictive adaptive response in *B. anynana* [37], with temperature experienced during final stage of larval development inducing the expression of alternative seasonal morphs.

The most noticeable differences between wet season form (WSF) and dry season form (DSF) adults of *B. anynana*, and those of many other mycalesines, are the colour patterns on the ventral wing surfaces of the adults which are exposed to predators when the butterflies are at rest [38]. In *B. anynana*, these differences are strongly associated with the alternative seasonal strategies to avoid predation [39, 40]. In the laboratory, the seasonal forms of *B. anynana* can be induced by the temperature experienced during the critical period of pre-adult development [41], with low and high temperatures inducing DSF-like and WSF-like phenotypes, respectively. Experiments with this model organism have revealed temperature-induced adaptive plasticity of behavioural [42], physiological [43] and life-history traits [44]. In the case of *B. anynana*, ecdysteroid hormone dynamics during the late-larval and pupal stages regulate development time [45] and mediate the synchronized phenotypic responses of a wide suite of morphological and life history traits, such as the formation of wing pattern elements [46–48], starvation resistance and female fecundity [49].

Closely related species of mycalesine butterflies, or even populations belonging to a single species, may be distributed over geographic areas with strikingly different seasonal environments [50, 51]. Seasonal forms have been described for many mycalesine species [38, 52, 53], however, the environmental cues inducing the expression of the alternative developmental pathways are less well understood for mycalesine species other than *B. anynana* [54]. Species inhabiting rainforests may express significant variation in wing pattern elements without being exposed to substantial seasonal variation in temperature [36]. Furthermore, in many tropical environments around the equator an increase in ambient temperature is associated with periods of limited precipitation [54]. Hence, in these environments the development of a WSF phenotype in response to a rapid rise in temperature would represent a non-adaptive response to the forthcoming environment.

Here, we present a comparative study on temperature-induced developmental plasticity across five mycalesine species and examine how patterns of developmental plasticity and potential local adaptation vary across closely related species. Multiple thermal environments were included in the laboratory experiments to enable a full analysis of the shape of the reaction norms for a wide suite of morphological and life-history traits. The species represent a broad range of ecological conditions in Africa, Madagascar and Asia, and their plastic responses are expected to have evolved in response to different ecological contexts with divergent selective pressures.

In this study, we investigate the potential for trait-independent responses by comparing how plastic traits are correlated within and across species, some of which have evolved in response to differing regimes of habitat seasonality. One of the African mycalesine species, *Bicyclus safitza* [Westwood, 1850], was collected in an environment where temperature is no longer a reliable predictor of forthcoming environmental conditions. The two Asian species, *Mycalesis mineus* (Linnaeus, 1758) and *Mycalesis perseoides* (Moore, 1892), were collected in an environment in which ambient temperature and rainfall are negatively correlated. Among these species, traits which can become uncoupled from the integrated phenotypic response to developmental temperature are predicted to show divergent correlative patterns as a result of differing responses to directional selection. Changes in reaction norm slope or shape are predicted to be greatest for wing pattern elements, and even inverted responses to developmental temperature may have evolved in the species which inhabit environments where a decrease in temperature is associated the onset of the wet season. Selection pressure on life history traits, such as total body size or allocation of mass to abdomen, is not expected to vary considerably across habitats and the reaction norms of these traits are therefore predicted to be more constrained compared to the morphological traits (i.e., wing pattern elements).

Finally, during the study we paid special attention to the patterns of developmental plasticity in *Heteropsis iboina* (Ward, 1870), a species that inhabits secondary forests and woodlands in Madagascar [55]. While being exposed to alternating warm-wet and cool-dry conditions in the wild, this species is known to have cryptic DSF-like ventral wing patterns throughout the year [55]. This suggests that, regardless of being exposed to a seasonal regime which is very similar to that of *B. anynana*, a more robust phenotype may have evolved in this species.

## Methods

### Butterflies and experimental design

The butterflies used in this study originated from stock populations founded between 2011 and 2013. The laboratory populations of *B. safitza* and *H. iboina* were established from eggs collected in Semuliki National Park in Uganda and Andasibe-Mantadia National Park on Madagascar, respectively. The founders of the laboratory populations of the two Asian species, *M. mineus* and *M. perseoides*, were collected near the Khao Chong Nature Reserve, Thailand. At least ten gravid females contributed to each stock population. For this study we also re-analysed the life history data and examined the wing material from a reaction norm experiment conducted using *B. anynana*, another mycalesine species from the African mainland (Nkhata Bay, Malawi). Details of this experiment are given in Oostra et al. [44]. Climate data for all locations were obtained from the Climatic Research Unit (CRU) online database which provided mean monthly temperature and precipitation for a period of 109 years [56].

Larvae were reared on young wheat plants (*Triticum aestivum*) in climate-controlled chambers (Sanyo/Panasonic MLR-350H) at 70% relative humidity (RH) with a 12:12 L:D cycle. Individuals were randomly divided over four climate-controlled chambers (21 °C, 23 °C, 25 °C and 27 °C) within 1 day after hatching. Cohorts of 20 larvae per plant were kept inside a sleeve of gauze-like material to ensure non-stressful feeding densities. Sleeves were checked daily and fresh plants provided when needed, to ensure ad libitum feeding conditions. Pre-pupae were collected daily and after pupation the pupae were individually placed in transparent pots until they eclosed. One day after eclosion the adults were frozen to −20 °C, sexed and stored in small envelopes until further processing. In total, 1243 individuals were included in the temperature reaction norm experiments, with an addition of 358 individuals of *B. anynana*.

### Life-history traits

The larval development time (LDT) and pupal development time (PDT) of each individual were measured as the number of days between hatching of the egg and pupation and the number of days between the final moult and adult eclosion, respectively. Together these measurements give the total development time (DT) of each individual. All pupae were weighed to the nearest 0.1 mg within 24 h after pupation (Fisherbrand PS-60) and the individual growth rate (GR) was calculated by dividing the natural logarithm of the pupal weight (PW) by the LDT (see Gotthard et al. [57]). All adults were carefully dissected, initially by removing the wings and legs. Subsequently, the abdomen and thorax were separated, dried to constant mass (60 °C for 24 h) and weighed individually, yielding total dry mass (DM) as well as the relative allocation of adult body mass to the abdomen (RA). Total fat (triglyceride and free fatty acids) was extracted by incubating the dried body parts at room temperature in 2:1 (v/v) dichloromethane:methanol for 96 h, followed by drying and re-weighing of the body parts. Absolute fat content was calculated by subtracting the fat-free dry weight from the initial dry mass after which the relative fat content (FC) was computed by dividing absolute fat content by the initial dry mass [58].

### Wing pattern elements

**Fig. 1:**
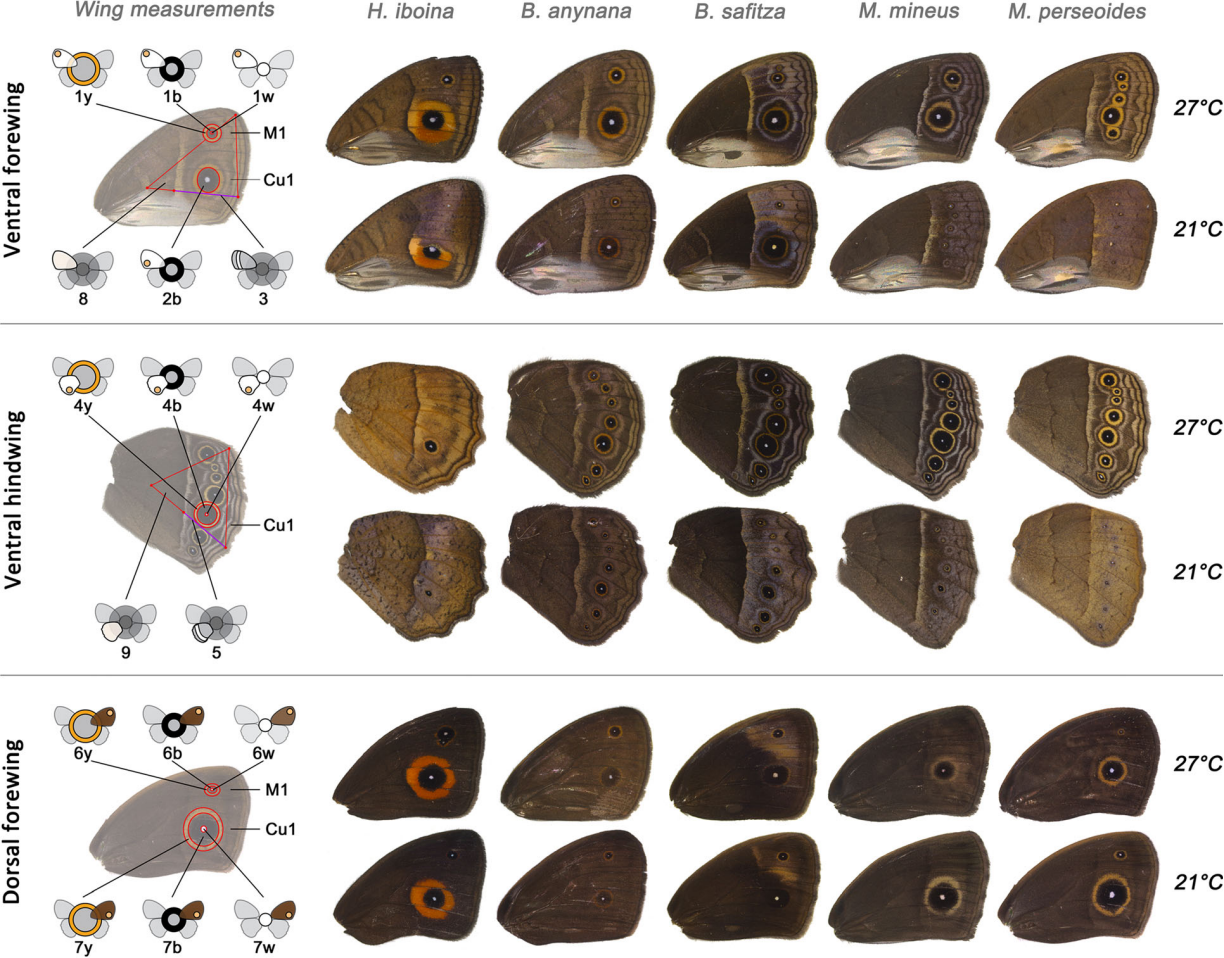
Wing traits measured in all specimens. The photos represent the typical phenotype of males reared at 27 °C (row 1, 3 and 5) and 21 °C (row 2, 4 and 6). Each column represents one species, from left to right: *H. iboina*, *B. anynana*, *B. safitza*, *M. mineus* and *M. perseoides*. For each individual, we obtained 17 wing measurements corresponding to four categories of traits: ventral eyespots (1,2,4), ventral bands (3,5), dorsal eyespots (6,7) and wing areas (8,9). Different letter codes were used to refer to the corresponding yellow rings (y), black discs (b) and white pupils (w). Icons provided by Manuel Marques-Pita and adjusted from Mateus et al. [47]

The ventral and dorsal surface of one hind- and one forewing of each individual were imaged using a Leica DFC495 digital camera coupled to a Leica M125 stereomicroscope and the photographs analysed with the image processing package Fiji [59]. Phenotypic traits on the wing surfaces can be measured with high repeatability with this image analysis system (overall R^2^ = 0.978 ± 0.023; see Additional file 1). On the ventral hindwing, the area of the yellow outer ring, the black inner-disc, and white focus of the eyespot in cell Cu1 were measured. In addition, the relative distance of the proximal edge of the median band along the second wing vein was taken as a measure of the width of the band, which is difficult to measure directly because of its indistinct distal edge (adjusted from Wijngaarden & Brakefield [60]). The measurements on the ventral forewing included the yellow, black and white areas of the eyespot in cell M1 as well as area of the black inner-disc of the larger eyespot in cell Cu1. On the dorsal forewing we measured the area of the yellow outer ring, the black disc, and white focus of the large eyespot in cell Cu1 and, if present, the smaller eyespot in cell M1. For all wings an area enclosed by three clear landmarks was used as a proxy of wing size. See Fig. 1 for a graphic overview of all measured wing pattern elements. References to wing veins and cells follow the Comstock-Needham system [61].

### Statistical analyses

All statistical analyses were performed with the R Statistical Package v 3.1.2 [62]. All development times were log-transformed to improve normality. The abdomen ratio and relative fat content were arcsine transformed and wing pattern elements were corrected for wing size. Two-way ANOVAs were used to analyse the effect of developmental temperature and sex on each phenotypic trait of interest for each species. Full models were fitted initially including temperature, sex, and their interaction as fixed factors, before removal of non-significant terms successively. Correlation matrices were used to demonstrate the integration of plastic responses, and to elucidate which traits are or have become uncoupled from the synchronized response to developmental temperature.

Nine wing-pattern measurements of the ventral wing surface (traits 1–5; see Fig. 1) were reduced using a principal component analysis, pooling data per species across the sexes. For each species, the first principal component (PC1) explained approximately 60% of the total variation and was strongly associated with the effect of the developmental temperature (Additional file 2). PC2 explained approximately 15% of the variation in each species and was correlated with the sex rather than seasonality. Therefore, only PC1 was used to analyse the general phenotypic response of the ventral wing surface (see supplementary material). Post hoc comparisons between specific levels of the factors were performed using Tukey’s honest significant differences (HSD) tests.

The relationship between developmental time and PC1 was explored by performing piecewise linear regressions, with development time as dependent variable and sex as covariate, using the ‘segmented’ package in R [63]. The existence of one or several inflection points and significant differences in slopes was tested using Davies’ tests [64, 65], after which the positions of the inflection points (i.e., the developmental thresholds) and 95% confidence intervals were estimated.

## Results

**Fig. 2:**
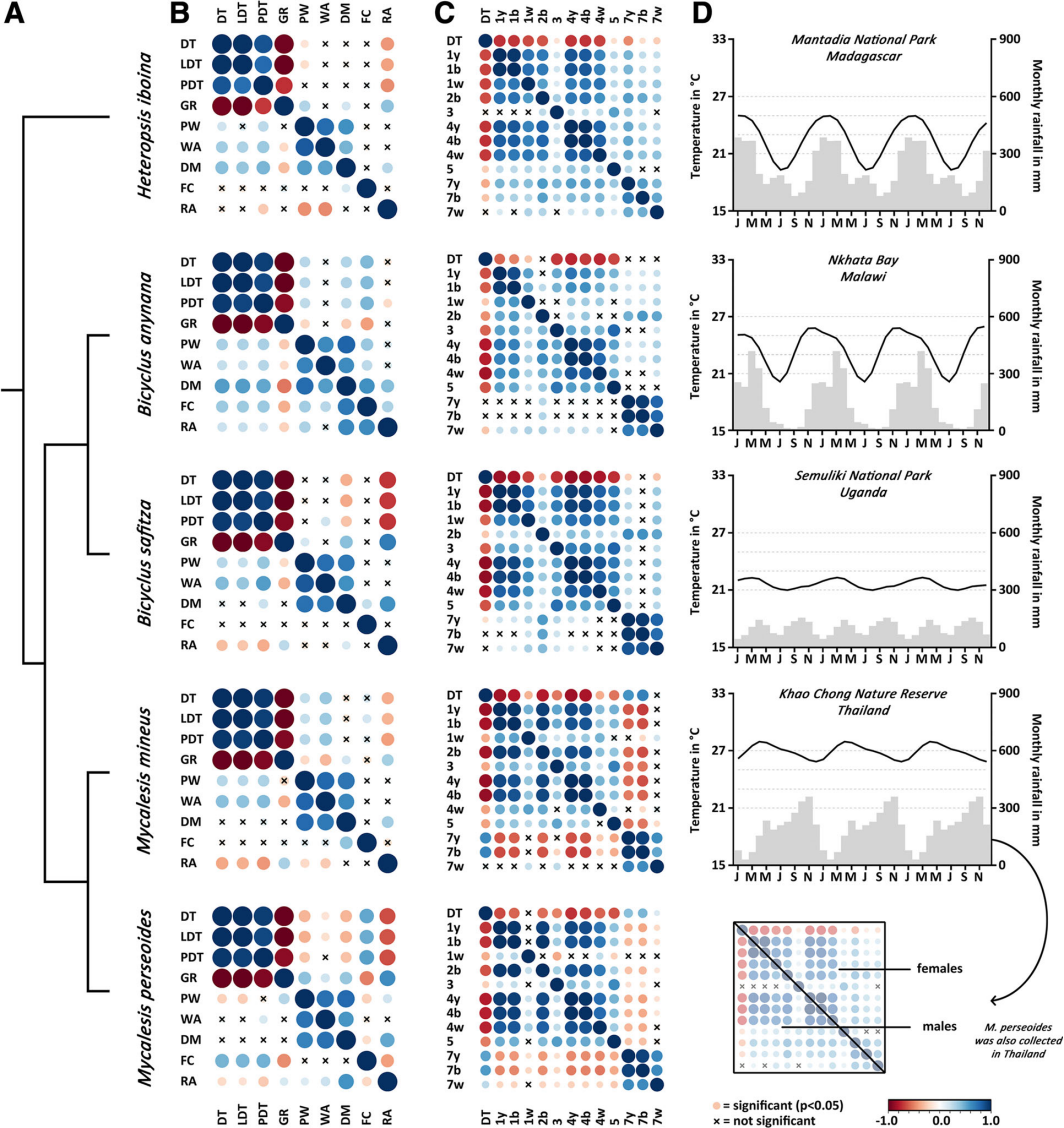
**a** Phylogenetic relationships among the species included in the study (see Aduse-Poku et al. [31]). **b** Correlations among life history traits within each species. Correlations in males are shown in cells in the lower left off-diagonal and correlations in females are given in the upper right off-diagonal. Positive correlations are denoted in blue and negative relationships in red. Non-significant correlations (*P* > 0.05) are indicated with an ‘X’. Abbreviations: total development time (DT), larval development time (LDT), pupal development time (PDT), growth rate (GR), pupal weight (PW), total wing area (WA), dry mass (DM), relative fat content (FC) and relative allocation of adult body mass to the abdomen (RA). **c** Correlations among wing pattern elements and development time within each species. The ventral eyespots (1,2,4), ventral bands (3,5) and dorsal eyespot (7) are numbered as in Fig. 1. **d** Climatic data from the locations in which the species were collected. Temperature (lines) and rainfall (bars) represent the average of the monthly mean for a period of 103 years

In all species studied here, most life history traits and wing pattern elements showed a significant response to developmental temperature. However, the shape of the reaction norms as well as the direction of the response varied significantly across species for several traits. Moreover, traits which showed significant responses to the temperature treatments were also highly correlated with developmental time in all species (Fig. 2). All minimum adequate models and reaction norm representations are presented in Additional files 3 and 4. Additional file 5 includes a comprehensive summary of all results, while we present the most salient results here.

### Developmental times and body size

In all species and both sexes, total egg to adult development time (DT) was strongly affected by developmental temperature (21 > 23 > 25 > 27 °C). Male larvae developed significantly faster than female larvae, while during the pupal stage, females of all species developed faster than males. The effect of the latter was small and protandry was present in all species at all developmental temperatures. Total wing area (WA), pupal weight (PW) and adult dry mass (DM) were significantly affected by developmental temperature in all species, with the exception of DM in *B. safitza*. The shapes of the reaction norms varied significantly among species and across these body size traits but overall we observed a consistent trend for larger individuals at lower temperatures (21 > 27 °C) for most species. Females were significantly larger than males across all temperatures and all species had significantly higher growth rates (GR) when reared at higher temperatures (see Additional file 3a–g).

### Fat content

**Fig. 3:**
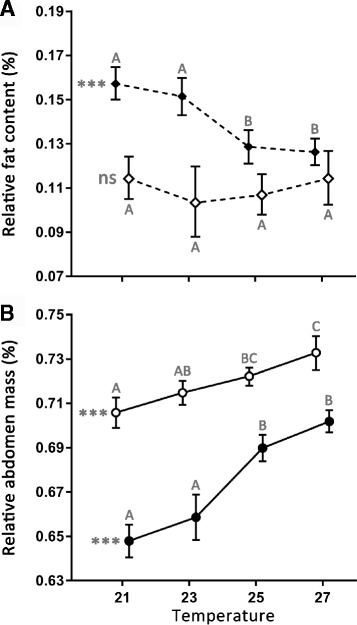
Effects of developmental temperature on relative fat content (**a**) and abdomen mass (**b**). The mean value is plotted as a function of temperature and these representations, called reaction norms, are the standard way of displaying plasticity. Panel **a** shows the relative fat content in males from *M. perseoides* (filled symbols) and *M. mineus*, (open symbols). Panel **b** represents the relative investment in abdomen mass by females from *H. iboina* (open symbols) and *B. safitza* (filled symbols). Statistical significance for effects of temperature on the life history trait is indicated to the left of each reaction norm: ns (non-significant) = *P* > 0.05 and *** = *p* < 0.001. Error bars represent 95% confidence intervals and significant differences across the temperature treatments (Tukey’s HSD, *p* < 0.05) are indicated by different letters, coding for each species separately. All minimum adequate models and reaction norm representations are presented in the supplementary materials

In *B. anynana* and *M. perseoides*, adult relative fat content (FC) decreased significantly with increasing developmental temperature in both sexes. In contrast, FC was not affected by temperature, nor correlated with developmental time, in either sex of *B. safitza*, *M. mineus* and *H. iboina* (Fig. 3a; Additional file 3h). In *B. anynana*, male FC did not change along the temperature gradient, with the exception of 27 °C where it was significantly lower compared to the other temperature treatments. Females of *B. anynana* developed the highest FC when reared at 21 °C (see also Oostra et al. [44]). In *M. perseoides*, the response to temperature was discontinuous with a developmental threshold between 23 °C and 25 °C for both females and males (Fig. 3a). In all species, FC of females was significantly lower than in males.

### Abdomen ratio

Individuals of all species allocated relatively more mass to the abdomen (RA) when reared at higher temperatures (Additional file 3i). In general, the effect of the temperature treatment on RA was particularly pronounced in females but, in most species, males responded in a similar way. Relationships between developmental temperature and phenotypic variation in RA ranged from linear responses, for example in females of *H. iboina* (Fig. 3b), to more discontinuous responses to temperature, as observed in females of *B. safitza*. The latter species allocated relatively more mass to the abdomen when reared at either of the two higher temperature treatments (Fig. 3b).

### Dorsal wing surface

**Fig. 4:**
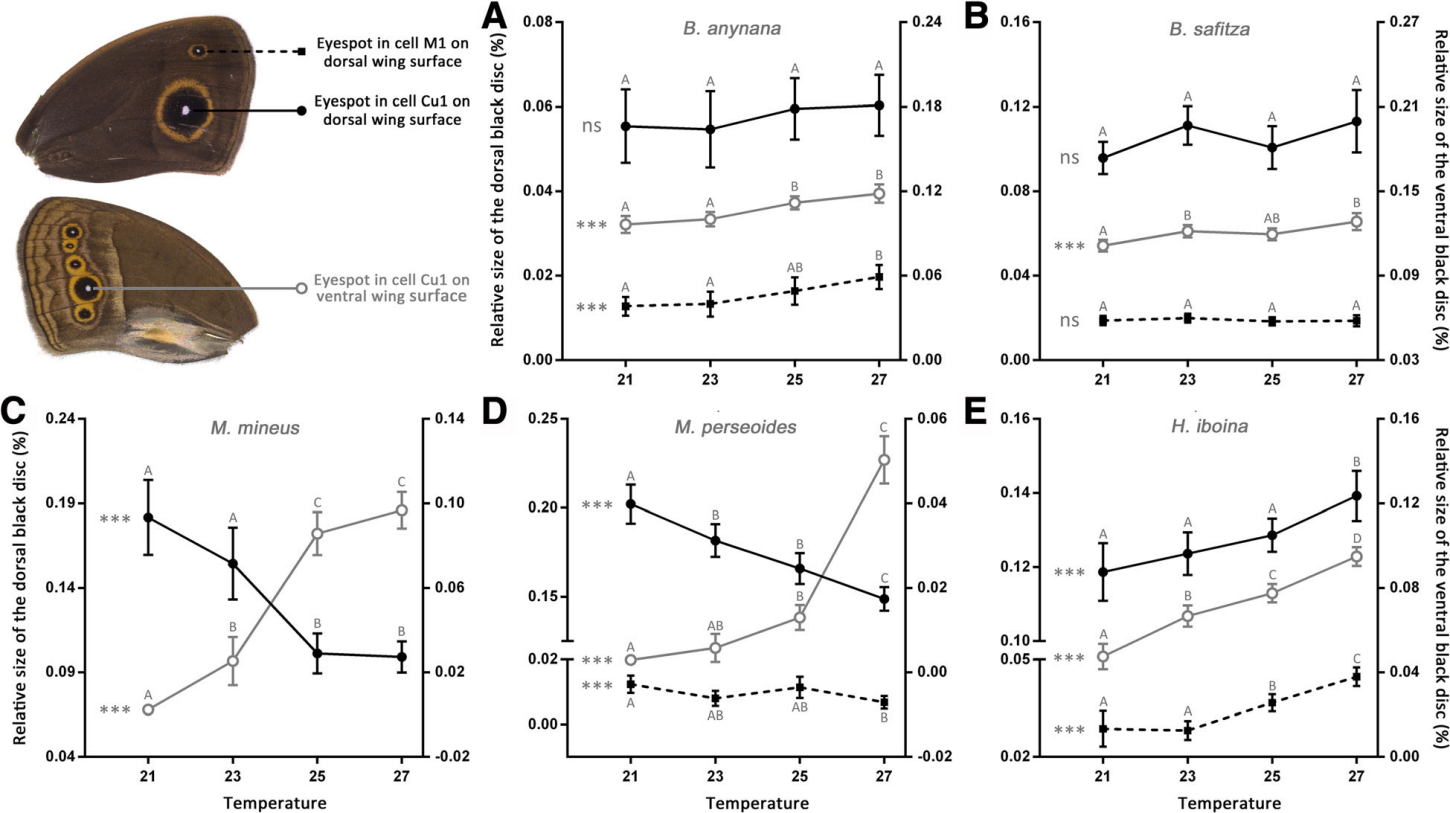
Effects of developmental temperature on the relative size of the black disc on both surfaces of the forewing for (**a**) *B. anynana*, (**b**) *B. safitza*, (**c**) *M. mineus*, (**d**) *M. perseoides* and (**e**) *H. iboina*. Data represent males but similar results were obtained for females. Filled symbols indicate the dorsal eyespots, with the solid line representing the black disc of the large eyespot in cell Cu1 (element 7) and the dashed line the smaller eyespot in cell M1 (element 6). Both these traits are associated with the left hand axis. The open symbols and grey lines represent the black disc of the large eyespot on the ventral wing surface (element 2). These values are associated with the right hand axis. Statistical significance for effects of temperature on the wing trait is indicated to the left of each reaction norm: ns (non-significant) *P* > 0.05, * *p* < 0.05, ** *p* < 0.01, *** *p* < 0.001. Error bars represent 95% confidence intervals and significant differences across the temperature treatments (Tukey’s HSD, *p* < 0.05) are indicated by different letters, coding for each trait separately

The analyses of the wing pattern elements on the dorsal surface revealed significant species-specific responses to developmental temperature. In both *Bicyclus* species, *B. anynana* and *B. safitza*, the large eyespot in cell Cu1 is relatively insensitive to developmental temperature while in the other three species the same eyespot was strongly affected by the same treatment (Fig. 4). In *H. iboina*, dorsal eyespot size increased almost linearly with increasing temperature, yielding large eyespots when reared at high temperatures. In contrast, in both *Mycalesis* species, *M. mineus* and *M. perseoides*, this pattern was reversed, showing a significant decrease in dorsal eyespot size with increasing temperatures; hence these species had small dorsal eyespots when reared at high temperatures (Fig. 4d and e). Similar responses were obtained for the small eyespot on the same wing surface with the exception that in *B. anynana* this eyespot was significantly affected by developmental temperature and became larger with increasing temperature (Fig. 4a).

### Ventral wing surface

**Fig. 5:**
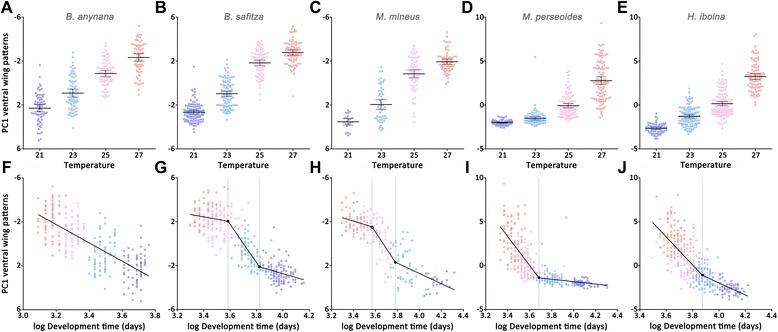
Effects of developmental temperature (**a**-**e**) and development time (**f**-**j**) on the first principal component (PC1) of nine ventral wing pattern elements, explaining about 60% of the variation in eyespot and band size in each species. Coloured dots represent the values for individuals reared at 21 °C (*blue*), 23 °C (light-blue), 25 °C (*pink*) and 27 °C (*red*). In graph **f**, a linear regression line was fitted and explained about 55% of the variation. In graphs **g-j**, piecewise linear regressions were fitted and explained over 60% of the variation in all species. The filled symbols indicate the significant inflection points, the secondary inflection point in *M. mineus*, indicated by an open symbol, was not significant

Most wing pattern elements on the ventral surface were strongly affected by the developmental temperature in all species, with higher temperatures yielding more conspicuous patterns (Fig. 5a–e). In *B. anynana*, the ventral wing patterns (PC1) responded almost linearly to developmental temperature, resulting in intermediate phenotypes at intermediate temperatures. In contrast, the relationship between the temperature and phenotypic gradient was non-linear in the other four species. The ventral wing patterns of *B. safitza* and *M. mineus* increased dramatically between 23 °C and 25 °C. As a consequence, in these two species, individuals reared at the extremes of the temperature gradient showed a very close phenotypic resemblance. In *M. perseoides* and *H. iboina*, the ventral wing patterns responded in an almost exponential manner to the temperature treatment, with a sharp increase between the two highest experimental temperatures. In addition, ventral wing patterns elements which demonstrated a clear response to variation in temperature were also significantly correlated with developmental time in all species (Fig. 2), such that faster developing individuals yield more conspicuous patterns.

### Developmental thresholds

The relationship between the ventral wing patterns (PC1) and developmental time mirrored the shape of the reaction norms, and we used this relationship to determine the presence of developmental thresholds for PC1 in these species (Fig. 5f–j). The variation in the ventral wing pattern elements of *B. anynana* correlates linearly with variation in developmental time, while in all other species significant inflection points were detected. In *H. iboina*, the relationship between PC1 and development time has a significant change in slope around a single inflection point (Davies’ test for a change in the slope, *p* < 0.001) at 48.2 days (95% CI: 45.1–51.5). In *M. perseoides* we also detected a single inflection point (*p* < 0.001) at 39.8 days (95% CI: 38.1–41.6). The relationship between PC1 and development time followed an S-shaped trajectory in *B. safitza* and had significant changes in the slope around two inflection points (both *p* < 0.05). Only a short developmental period between the first (36.0; CI: 34.2–37.9) and the second inflection point (45.7; CI: 43.7–47.9) leads to the induction of intermediate phenotypes. A similar pattern was found for *M. mineus*, with an initial significant developmental threshold (35.3; CI: 33.1–37.7). However, in this species, the secondary inflection point, predicted at 44.1, was not significant (*P* = 0.28).

## Discussion

### Integrated responses to environmental stress are conserved

All mycalesine species included in this study demonstrated temperature-induced developmental plasticity. In general, individuals reared at lower temperatures not only develop slower but are also larger and have lower growth rates, which seems to be the norm for ectotherms [66]. As predicted, correlative patterns among these life history traits, as well as reaction norms shapes, were highly conserved across all species. Moreover, despite the fact that the species represent considerable periods of independent evolution and occurrence in disparate environments, we also demonstrate that all species invest less in reproduction (i.e., reduced allocation of body mass to the abdomen) and possess less conspicuous ventral wing patterns when reared at lower temperatures. Interestingly, the responses to developmental temperature in relative abdomen mass and ventral wing patterning are known to be regulated by ecdysteroid signalling in *B. anynana*. Ecdysteroid injections during the early pupal stage accelerate development, increase body mass allocation to abdomen in females [49] and induce the expression of more conspicuous ventral wing patterns [47, 48]. In *B. anynana*, several other life history traits co-vary with the traits examined here in their response to developmental temperature. For example, a higher resistance to starvation [43] and increased longevity in adults [67] are associated with exposure to lower developmental temperatures and respond in similar directions to manipulations of internal levels of ecdysone [49]. The evolution of this particular functional suite, in which the plastic responses of key traits are integrated to express a coordinated environmental response, is anticipated to be an adaptation to variability in environmental stress in *B. anynana*. The direction of the response to the temperature gradient was very similar across all species for the traits that make up the functional suite which suggests that the sharing of the same underlying hormonal regulator may be conserved within the mycalesine subtribe (see Additional file 5 for a summary table of the results).

### Correlated responses of independent traits

The presence of strong phenotypic correlations between traits does not necessarily imply that plastic responses are integrated and mediated by the same internal signals. The lack of response to temperature for relative fat content (FC) in *B. safitza*, *M. mineus* and *H. iboina* observed in this study could be interpreted as an example of a trait which has become uncoupled from the synchronized response to environmental variation in these species. Thus, while the responses to temperature are very similar for all traits of the functional suite in these five species, FC could respond independently to the selective forces experienced in novel environments. Such uncoupling may be because FC in these species is not regulated by pupal ecdysteroid dynamics, although ecdysteroid injections have been shown to induce higher abdominal fat content in *B. anynana* females [49]. Alternatively, it is also possible that FC does respond to temperature in *B. safitza*, *M. mineus* and *H. iboina*, but the threshold temperature at which FC is altered in these species may lie outside the range of temperatures used in this study.

In this context, one particularly intriguing result of this study is the variation in the plastic responses of the eyespots on the dorsal wing surface. In contrast to the ventral wing surface, where all pattern elements were highly correlated and responded in the same direction in all species, there were striking differences across species in the responses of the patterns on the dorsal wing surface to developmental temperature. In *H. iboina*, the size of the dorsal eyespot (wing pattern element 7; see Figs. 1 and 3) was positively correlated with the ventral wing patterns and increased with increasing temperature. In contrast, in both *Mycalesis* species, the dorsal eyespot size responded in the opposite direction to the same environmental gradient and this trait was negatively correlated with the patterns on the ventral wing surface. Furthermore, in these species the ventral and dorsal eyespots of same wing not only responded in opposite directions but also with very different dynamics (i.e., different reaction norm shapes; Fig. 4). Finally, in both *Bicyclus* species the same eyespot was largely insensitive to variation in developmental temperature, which confirms the results of previous studies on dorsal wing patterning in *B. anynana* [42, 68]. Interestingly, in this species, pattern elements on the dorsal wing surface do not respond to ecdysone titre manipulations [47]. This validates the idea that colour pattern formation on both wing surfaces is independent [69, 70] and suggests that the two developing tissues respond to different internal signals. In addition, these results demonstrate that antagonistic responses to the same inductive environmental cue can evolve independently among a group of closely related species.

### Eyespots and antipredator strategies

In *B. anynana*, wet season form adults (WSF) show conspicuous eyespots on the ventral wing surfaces, positioned close to the margin of the wing. These eyespots function to deflect the attacks of predators to regions of the body that are non-vital and, thus, increase adult survival and fitness [39, 40]. In contrast, dry season form adults (DSF) of *B. anynana* have virtually no ventral eyespots, relying on being cryptic when at rest against a background of leaf litter to evade predators [71]. During this study we paid special attention to the patterns of developmental plasticity in *H. iboina*. Unlike the other four species, this species from Madagascar has a cryptic DSF-like appearance throughout the year, and we hypothesised that, regardless of being exposed to substantial seasonal variation, a more robust and less plastic phenotype had evolved in this species. In contrast, we found that most life history traits and measured wing pattern elements showed a significant response to developmental temperature in *H. iboina*, demonstrating that the cryptic phenotype of this species is not achieved by a (complete) loss of plasticity for ventral wing patterns. Instead, crypsis seems to be maintained due to the effective absence of expression of several marginal eyespots on the ventral hindwing ‒ possibly via a shift of the reaction norm along the developmental environmental axis ‒ where the other four species demonstrate a pattern of serially repeated eyespots on the ventral hindwing (see Fig. 1). This may indicate that ventral deflective eyespots are less advantageous in this species. Interestingly, a ritualised display or ‘startling’ behaviour involving the sudden exposure of highly conspicuous eyespots on the dorsal surface has been observed frequently in *H. iboina* in the field (personal observations). Such displays have been shown to be effective in startling potential vertebrate predators to increase the probability of escape in numerous other species of Lepidoptera [72, 73]. The startle behaviour of *H. iboina* may be an alternative adaptation to avoid predation in the Malagasy forests.

In this context, for the two species from Asia, *Mycalesis mineus* and *Mycalesis perseoides*, which inhabit an environment in which ambient temperature and rainfall are negatively correlated, the integrated response of ventral wing patterns to developmental temperature could be maladaptive. Individuals with cryptic ventral wing patterns may only start to emerge in the early wet season when dead, brown leaf litter is expected to be less abundant. This suggests that the power of selection, favouring conspicuous ventral eyespots for deflection during the wet season, may be constrained by the integration of plastic responses in these species. Alternatively, these species may use environmental cues other than temperature to match their seasonal forms with the seasonality of the climate [54]. Remarkably, in both *Mycalesis* species the eyespots on the dorsal wing surface respond in the opposite direction to developmental temperature and individuals with large dorsal eyespots are expected to emerge during the wet season based on our experimental data. Alternative anti-predator behaviour could have evolved in these species in response to a lower evolvability of the ventral wing patterns. Our data indicate that it is now timely to assess how patterns of plasticity are related to the evolution of species-specific antipredator strategies.

### Developmental time and thresholds

Our results clearly indicate that rearing temperature alone can induce plastic responses in mycalesine butterflies, however little is known about the effect of the environmental complexity to which populations are exposed in the wild. In this study all the traits that showed a clear response to temperature were also highly correlated with developmental time. Based on observations from laboratory work with *B. anynana* it can be hypothesized that any environmental factor which influences the duration of pre-adult development could act as a morph-determining, predictive cue [71]. For example, food limitation during larval development [74] and a reduced nutritional quality of the diet [75, 76] are strongly associated with longer development times and the induction of more DSF-like phenotypes. In addition, populations which are exposed to daily fluctuations in temperature or thermoperiods during development have larger eyespots and complete pre-adult development faster [77].

By using development time as a proxy for environmental variation during pre-adult growth we detected clear developmental thresholds for the expression of ventral wing pattern elements in all the non-model-species. Whenever developmental time exceeded these thresholds, transition into the alternative seasonal morph occurred. The pattern of *B. safitza* and *M. mineus* followed a trajectory which is typical of polyphenism. The induction of intermediate phenotypes, which may have lower fitness depending on the natural environment, only occurred during a restricted range of developmental times. In *H. iboina* and *M. perseoides*, we detected single inflection points in the developmental reaction norms. However, a second inflection point and levelling off of the phenotypic response may lie outside the temperature range used in this study. Alternatively, this phenomenon could be related to selection regimes and patterns of local adaptation in the wild. For example, by shifting the developmental threshold outside the boundaries of the typical temperature gradient, species will yield a more robust phenotype in the natural environment without losing the ability to express phenotypic plasticity when exposed to novel environments (see also Ghalambor et al. [13]). In *B. anynana*, the variation in the ventral wing pattern elements correlated linearly with variation in developmental time and intermediate phenotypes are induced at intermediate temperatures. However, extensive longitudinal surveys in a seasonal environment in Malawi suggest that such intermediate temperatures may not persist for sufficient time during key periods of larval development to induce (many) intermediate adult phenotypes in the wild [78].

## Conclusions

This study aimed to explore further the role of plasticity in driving patterns of evolutionary diversification; does it facilitate some directions of phenotypic evolution whilst also tending to limit change in other directions? Our results provide some support for the latter notion. Thus, we demonstrate that the integrated phenotypic plasticity of a broad suite of morphological and life history traits is shared among species from three geographically independent lineages of mycalesine butterflies, but at the same time, there are examples of evolutionary change where independent traits show different, ‘uncoupled’ patterns of reaction norm evolution. More extensive studies, made in combination with assays of phenotypic plasticity in the wild and analysed in a comparative phylogenetic framework, will be needed to examine more rigorously how phenotypic plasticity evolves among related species, and how conserved patterns of plasticity, based as here in a shared physiology, may influence subsequent patterns of reaction norm evolution.

## Additional files

Additional file 1: Repeatability estimates for wing pattern elements. (PDF 17 kb)
Additional file 2: Results of the principal component analyses (PCA) for wing pattern elements. (PDF 22 kb)
Additional file 3: Reaction norm graphs and minimum adequate models for all life history traits. (PDF 702 kb)
Additional file 4: Reaction norm graphs and minimum adequate models for all wing pattern elements. (PDF 1359 kb)
Additional file 5: Summary table of the plastic responses to developmental temperature in mycalesine butterflies. (PDF 29 kb)
Additional file 6: Raw dataset used in this study. (XLSX 581 kb)

## Abbreviations

DM: Dry mass
DSF: Dry season form
DT: Total development time
FC: Relative fat content
GR: Growth rate
LDT: Larval development time
PDT: Pupal development time
PW: Pupal weight
RA: Relative allocation of adult body mass to the abdomen
WA: Total wing area
WSF: Wet season form
