# Supplementary material for: Conserved patterns of integrated developmental plasticity in a group of polyphenic tropical butterflies
Source: BMC Evol Biol. 2017 Feb 27;17:59. doi: 10.1186/s12862-017-0907-1 (PMC5327525; doi:10.1186/s12862-017-0907-1)
Supplement: Additional file 1: — Repeatability estimates for wing pattern elements. (PDF 17 kb) [file 12862_2017_907_MOESM1_ESM.pdf]

Additional file 1: The repeatability for the wing pattern elements was estimated using 114 individuals, representing all species and both sexes. m_1 and m_2 represent the same individual being processed twice by our imaging and analysis platform. m_2 and m_3 represent the same image, being analysed twice. Imaging and analysis were conducted by four different researchers and yield high levels of repeatability (R^2). Numbers refer to figure 1 in the main text.

		m_1 vs m_2		*m_2 vs m_3*	
**Trait** (wing pattern element)		**Regression Coefficient**	**R-squared**	**Regression Coefficient**	**R-squared**
VFW WhiteArea M1	(1w)	0.884	0.894	0.968	0.876
VFW BlackArea M1	(1b)	1.002	0.992	0.993	0.990
VFW YellowArea M1	(1y)	0.990	0.990	0.990	0.993
VFW BlackArea Cu1	(2)	0.999	0.992	0.996	0.994
VFW Band	(3)	0.964	0.963	0.980	0.966
VHW Band	(5)	0.997	0.990	0.995	0.989
DFW WhiteArea M1	(6w)	1.019	0.983	0.996	0.990
DFW BlackArea M1	(6b)	1.015	0.998	1.015	0.998
DFW YellowArea M1	(6y)	1.012	0.998	0.997	0.998
DFW WhiteArea Cu1	(7w)	0.973	0.953	0.988	0.948
DFW BlackArea Cu1	(7b)	0.989	0.986	1.008	0.988
DFW YellowArea Cu1	(7y)	0.989	0.991	1.003	0.995
VFW HeronsFormula	(8)	0.998	0.984	0.993	0.994
VFW Xto2nd		0.986	0.976	0.990	0.983
VFW 2to7th		1.021	0.975	0.997	0.996
VFW Xto7th		0.981	0.973	0.998	0.985
VHW HeronsFormula	(9)	0.985	0.987	0.986	0.993
VHW Xto2nd		0.986	0.976	0.990	0.983
VHW 2to7th		1.006	0.985	1.004	0.993
VHW Xto7th		0.977	0.975	0.977	0.979
Overall (mean)		0.989	0.978	0.993	0.981
