# Supplementary material for: Conserved patterns of integrated developmental plasticity in a group of polyphenic tropical butterflies
Source: BMC Evol Biol. 2017 Feb 27;17:59. doi: 10.1186/s12862-017-0907-1 (PMC5327525; doi:10.1186/s12862-017-0907-1)
Supplement: Additional file 2: — Results of the principal component analyses (PCA) for wing pattern elements. (PDF 22 kb) [file 12862_2017_907_MOESM2_ESM.pdf]

Additional file 2: Principal component analyses of reaction norm data. Table gives the first two principal components (PC1 and PC2) of nine wing pattern measurements and the percentage of variation explained by each principal component. PC1 explained over 55% of the variation in the data in all species and was associated with seasonality.

		B. anynana		*B. safitza*		*M. mineus*		*M. perseoides*		*H. iboina*	
**Trait**	**Element**	**PC1**	**PC2**	**PC1**	**PC2**	**PC1**	**PC2**	**PC1**	**PC2**	**PC1**	**PC2**
VFW WhiteArea M1	(1w)	-0.247	0.130	0.320	0.004	-0.236	-0.205	0.107	-0.311	-0.343	0.085
VFW BlackArea M1	(1b)	-0.317	0.503	0.383	0.161	-0.410	-0.024	0.412	-0.016	-0.389	0.106
VFW YellowArea M1	(1y)	-0.374	0.342	0.393	0.090	-0.417	0.025	0.413	0.015	-0.389	0.076
VFW BlackArea Cu1	(2b)	-0.243	-0.085	0.122	0.636	-0.409	-0.039	0.403	-0.028	-0.339	-0.089
VFW Band	(3)	-0.299	-0.548	0.265	-0.525	-0.179	0.625	-0.027	0.682	-0.050	-0.754
VHW WhiteArea Cu1	(4w)	-0.371	-0.124	0.357	0.001	-0.222	-0.421	0.380	-0.085	-0.357	-0.009
VHW BlackArea Cu1	(4b)	-0.390	0.127	0.386	0.168	-0.396	-0.119	0.407	0.027	-0.393	0.093
VHW YellowArea Cu1	(4y)	-0.411	0.049	0.394	0.098	-0.406	-0.044	0.409	0.064	-0.397	0.075
VHW Band	(5)	-0.303	-0.522	0.283	-0.498	-0.181	0.609	0.089	0.652	-0.153	-0.621
Proportion of Variance		0.548	0.144	0.665	0.152	0.600	0.190	0.623	0.205	0.631	0.153
Cumulative Proportion		0.548	0.692	0.665	0.817	0.600	0.790	0.623	0.827	0.631	0.784
