# Supplementary material for: Conserved patterns of integrated developmental plasticity in a group of polyphenic tropical butterflies
Source: BMC Evol Biol. 2017 Feb 27;17:59. doi: 10.1186/s12862-017-0907-1 (PMC5327525; doi:10.1186/s12862-017-0907-1)
Supplement: Additional file 3: — Reaction norm graphs and minimum adequate models for all life history traits. (PDF 702 kb) [file 12862_2017_907_MOESM3_ESM.pdf]

Additional file 3: Reaction norm graphs and minimum adequate models for all life history traits.

3A: Total development time

Effects of developmental temperature on total development time (d) in *ANY*, *SAF*, *MIN*, *PER* and *IBO*. The mean value is plotted as a function of temperature and error bars represent 95% confidence intervals. Females and males are represented by the solid and dotted lines, respectively.

Minimum adequate models of the effect of developmental temperature and sex on the same trait. Statistical significance is indicated as: * P <0.05, ** P <0.01, *** P <0.001.

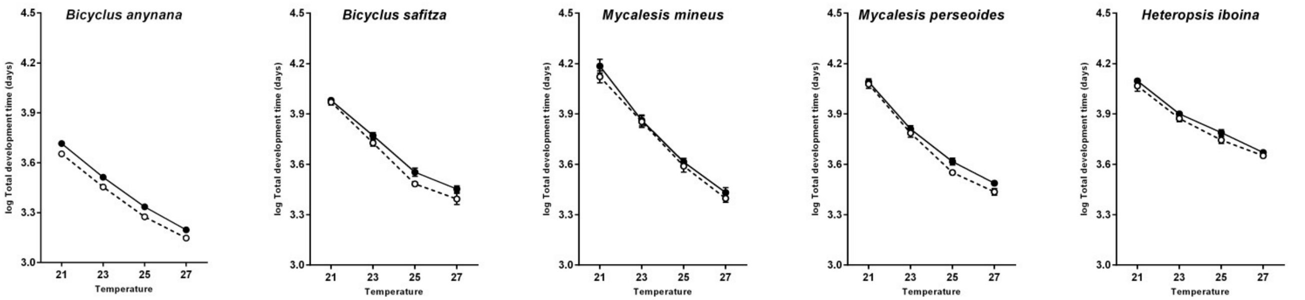

Trait	(transformation)	Fixed effects	F	df	df	P	F	df	df	P	F	df	df	P	F	df	df	P	F	df	df	P
Total development time (d)	(log)	Temperature	3535.0	3	272	***	1274.5	3	350	***	650.1	3	202	***	1361.4	3	353	***	596.9	3	393	***
		Sex	238.2	1	272	***	37.4	1	350	***	6.5	1	202	*	32.6	1	353	***	20.9	1	393	***
		Temperature x Sex					3.6	3	350	*					2.6	3	353	*				

3B: Larval development time

Effects of developmental temperature on larval development time (d) in *ANY*, *SAF*, *MIN*, *PER* and *IBO*. The mean value is plotted as a function of temperature and error bars represent 95% confidence intervals. Females and males are represented by the solid and dotted lines, respectively.

Minimum adequate models of the effect of developmental temperature and sex on the same trait. Statistical significance is indicated as: * P <0.05, ** P <0.01, *** P <0.001.

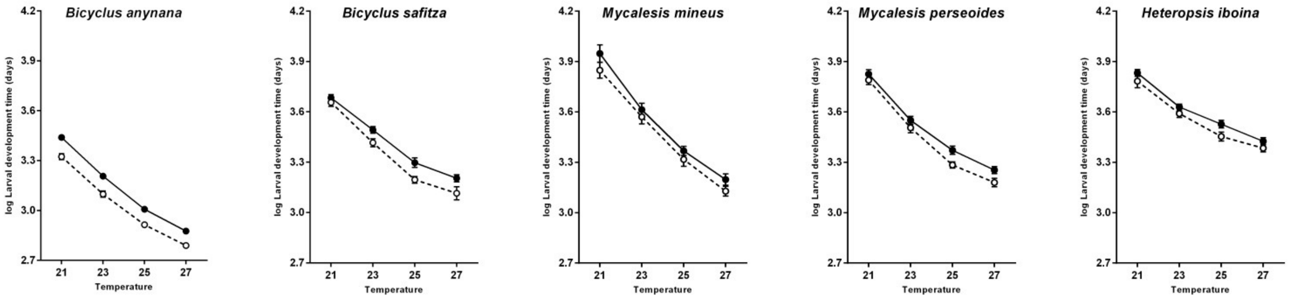

Trait	(transformation)	Fixed effects	F	df	df	P	F	df	df	P	F	df	df	P	F	df	df	P	F	df	df	P
Larval development time (d)	(log)	Temperature	1939.0	3	272	***	758.5	3	350	***	444.6	3	202	***	816.8	3	356	***	353.0	3	393	***
		Sex	348.0	1	272	***	69.9	1	350	***	21.3	1	202	***	51.6	1	356	***	36.5	1	393	***
		Temperature x Sex					3.9	3	350	*												

3C: Pupal development time

Effects of developmental temperature on pupal development time (d) in *ANY*, *SAF*, *MIN*, *PER* and *IBO*. The mean value is plotted as a function of temperature and error bars represent 95% confidence intervals. Females and males are represented by the solid and dotted lines, respectively.

Minimum adequate models of the effect of developmental temperature and sex on the same trait. Statistical significance is indicated as: * P <0.05, ** P <0.01, *** P <0.001.

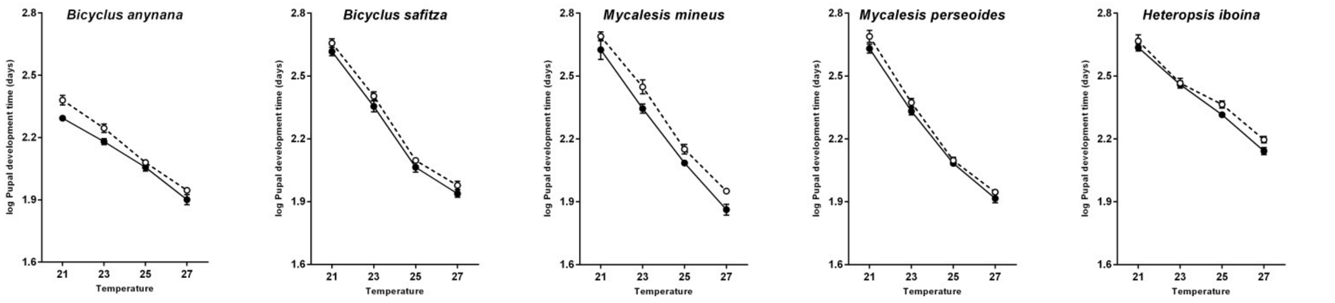

Trait	(transformation)	Fixed effects	F	df	df	P	F	df	df	P	F	df	df	P	F	df	df	P	F	df	df	P
Pupal development time (d)	(log)	Temperature	941.7	3	269	***	1765.2	3	353	***	1224.8	3	202	***	2514.4	3	356	***	833.0	3	390	***
		Sex	89.2	1	269	***	30.3	1	353	***	95.8	1	202	***	30.2	1	356	***	30.6	1	390	***
		Temperature x Sex	5.5	3	269	**													3.0	3	390	*

3D: Growth rate

Effects of developmental temperature on growth rate (mg/d) in *ANY*, *SAF*, *MIN*, *PER* and *IBO*. The mean value is plotted as a function of temperature and error bars represent 95% confidence intervals. Females and males are represented by the solid and dotted lines, respectively.

Minimum adequate models of the effect of developmental temperature and sex on the same trait. Statistical significance is indicated as: * P <0.05, ** P <0.01, *** P <0.001.

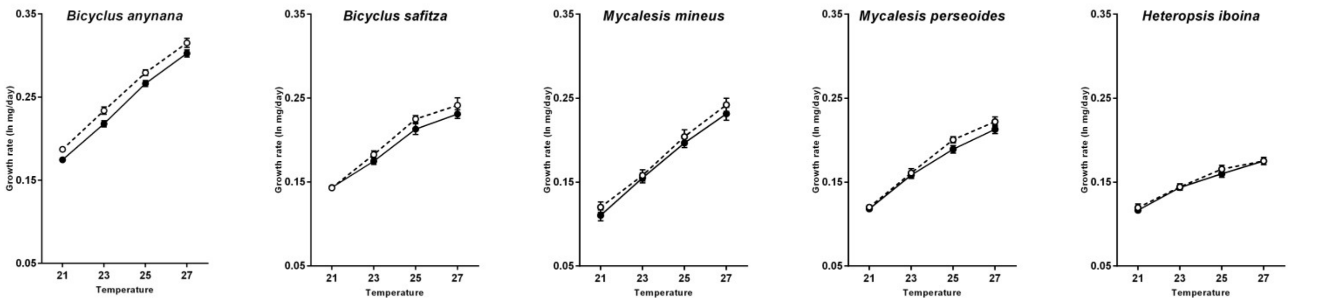

Trait	(transformation)	Fixed effects	F	df	df	P	F	df	df	P	F	df	df	P	F	df	df	P	F	df	df	P
Growth rate (mg/d)	log(mg) / d	Temperature	1630.2	3	269	***	628.8	3	350	***	334.7	3	199	***	646.4	3	349	***	243.5	3	390	***
		Sex	95.5	1	269	***	16.8	1	350	***	8.8	1	199	**	16.5	1	349	***	2.8	1	390	0.09
		Temperature x Sex					2.9	3	350	*												

3E: Pupal mass

Effects of developmental temperature on pupal mass (mg) in *ANY*, *SAF*, *MIN*, *PER* and *IBO*. The mean value is plotted as a function of temperature and error bars represent 95% confidence intervals. Females and males are represented by the solid and dotted lines, respectively.

Minimum adequate models of the effect of developmental temperature and sex on the same trait. Statistical significance is indicated as: * P <0.05, ** P <0.01, *** P <0.001.

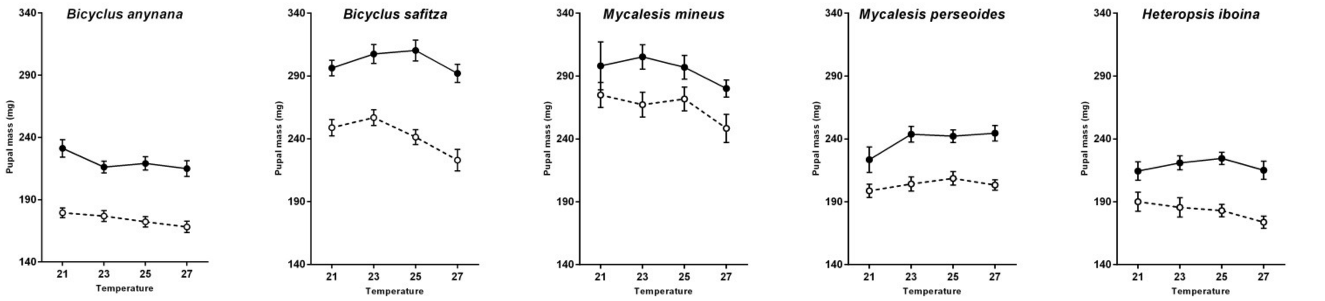

Trait	(transformation)	Fixed effects	F	df	df	P	F	df	df	P	F	df	df	P	F	df	df	P	F	df	df	P
Pupal mass (mg)		Temperature	10.0	3	272	***	11.6	3	350	***	9.3	3	202	***	12.3	3	349	***	9.1	3	393	***
		Sex	653.8	1	272	***	536.3	1	350	***	72.8	1	202	***	293.7	1	349	***	271.0	1	393	***
		Temperature x Sex					5.4	3	350	**					3.0	3	349	*				

3F: Total wing area

Effects of developmental temperature on total wing area (mm^2) in *ANY*, *SAF*, *MIN*, *PER* and *IBO*. The mean value is plotted as a function of temperature and error bars represent 95% confidence intervals. Females and males are represented by the solid and dotted lines, respectively.

Minimum adequate models of the effect of developmental temperature and sex on the same trait. Statistical significance is indicated as: * P <0.05, ** P <0.01, *** P <0.001.

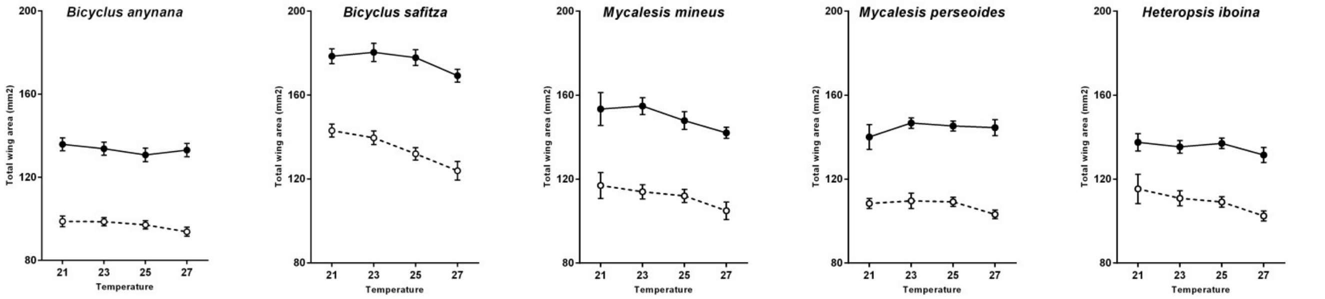

Trait	(transformation)	Fixed effects	F	df	df	P	F	df	df	P	F	df	df	P	F	df	df	P	F	df	df	P
Total wing area (mm^2)		Temperature	3.5	3	272	*	22.7	3	350	***	9.2	3	200	***	12.5	3	353	***	21.1	3	393	***
		Sex	1431.8	1	272	***	1041.6	1	350	***	707.2	1	200	***	1155.7	1	353	***	482.0	1	393	***
		Temperature x Sex					3.7	3	350	*					3.2	3	353	*				

3G: Adult dry mass

Effects of developmental temperature on adult dry mass (mg) in *ANY*, *SAF*, *MIN*, *PER* and *IBO*. The mean value is plotted as a function of temperature and error bars represent 95% confidence intervals. Females and males are represented by the solid and dotted lines, respectively.

Minimum adequate models of the effect of developmental temperature and sex on the same trait. Statistical significance is indicated as: * P <0.05, ** P <0.01, *** P <0.001.

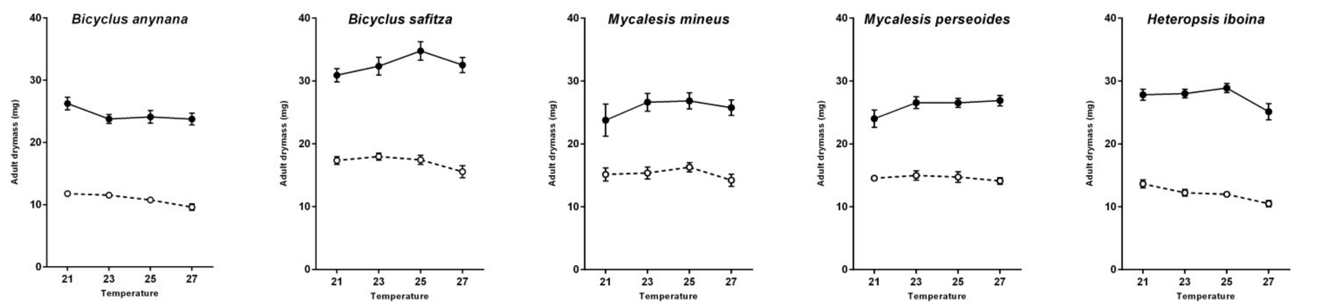

Trait	(transformation)	Fixed effects	F	df	df	P	F	df	df	P	F	df	df	P	F	df	df	P	F	df	df	P
Adult dry mass (mg)		Temperature	14.1	3	265	***	1.3	3	347	0.27	4.9	3	200	**	13.5	3	350	***	72.3	3	371	***
		Sex	2923.6	1	265	***	1637.0	1	347	***	575.2	1	200	***	1677.7	1	350	***	3006.2	1	371	***
		Temperature x Sex	4.0	3	265	**	6.1	3	347	***					5.6	3	350	***	4.5	3	371	*

3H: Adult fat content

Effects of developmental temperature on adult fat content (%) in *ANY*, *SAF*, *MIN*, *PER* and *IBO*. The mean value is plotted as a function of temperature and error bars represent 95% confidence intervals. Females and males are represented by the solid and dotted lines, respectively.

Minimum adequate models of the effect of developmental temperature and sex on the same trait. Statistical significance is indicated as: * P <0.05, ** P <0.01, *** P <0.001.

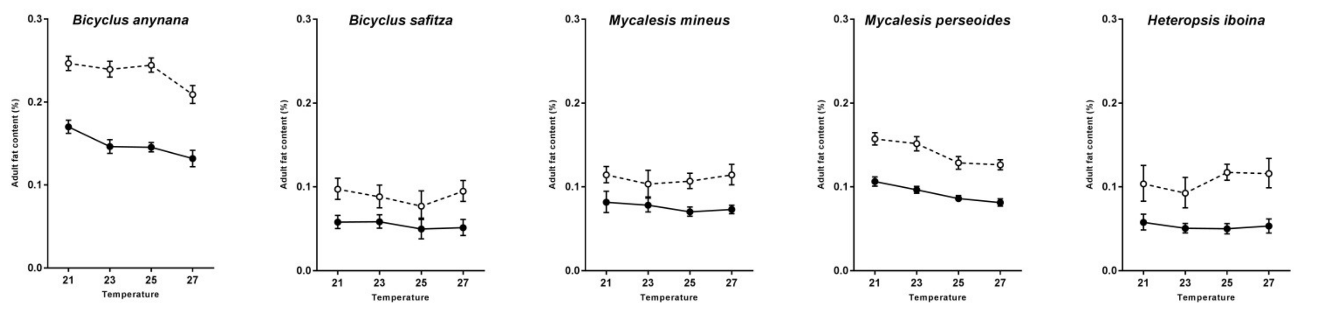

Trait	(transformation)	Fixed effects	F	df	df	P	F	df	df	P	F	df	df	P	F	df	df	P	F	df	df	P
Adult fat content (%)		Temperature	24.1	3	264	***	1.4	3	345	0.25	1.4	3	199	0.25	42.1	3	352	***	4.6	3	360	**
		Sex	739.8	1	264	***	70.6	1	345	***	100.7	1	199	***	525.6	1	352	***	195.0	1	360	***
		Temperature x Sex	3.1	3	264	*																

3I: Abdomen ratio

Effects of developmental temperature on the abdomen ratio (%) in *ANY*, *SAF*, *MIN*, *PER* and *IBO*. The mean value is plotted as a function of temperature and error bars represent 95% confidence intervals. Females and males are represented by the solid and dotted lines, respectively.

Minimum adequate models of the effect of developmental temperature and sex on the same trait. Statistical significance is indicated as: * P <0.05, ** P <0.01, *** P <0.001.

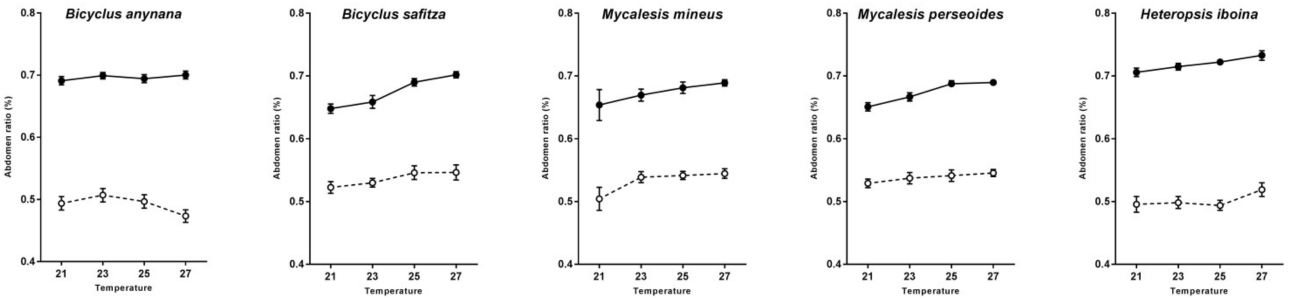

Trait	(transformation)	Fixed effects	F	df	df	P	F	df	df	P	F	df	df	P	F	df	df	P	F	df	df	P
Abdomen ratio (%)		Temperature	7.3	3	265	***	41.9	3	347	***	20.9	3	200	***	40.1	3	350	***	7.1	3	374	***
		Sex	4198.2	1	265	***	1915.8	1	347	***	1537.8	1	200	***	3286.6	1	350	***	6288.4	1	374	***
		Temperature x Sex	6.1	3	265	***	5.9	3	347	**					6.5	3	350	***				
