# Supplementary material for: Conserved patterns of integrated developmental plasticity in a group of polyphenic tropical butterflies
Source: BMC Evol Biol. 2017 Feb 27;17:59. doi: 10.1186/s12862-017-0907-1 (PMC5327525; doi:10.1186/s12862-017-0907-1)
Supplement: Additional file 4: — Reaction norm graphs and minimum adequate models for all wing pattern elements. (PDF 1359 kb) [file 12862_2017_907_MOESM4_ESM.pdf]

Additional file 4: Reaction norm graphs and minimum adequate models for all wing pattern elements.

4A: VFW WhiteArea M1 (1w)

Effects of developmental temperature on wing pattern element 1w in *ANY*, *SAF*, *MIN*, *PER* and *IBO* (see figure 1 in main text). The mean value is plotted as a function of temperature and error bars represent 95% confidence intervals. Females and males are represented by the solid and dotted lines, respectively.

Minimum adequate models of the effect of developmental temperature and sex on the same trait. Statistical significance is indicated as: * P <0.05, ** P <0.01, *** P <0.001.

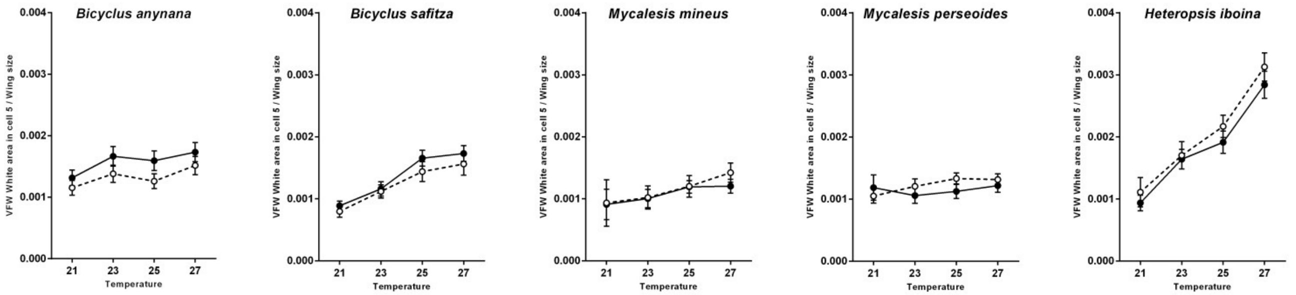

Trait	(transformation)	Fixed effects	F	df	df	P	F	df	df	P	F	df	df	P	F	df	df	P	F	df	df	P
VFW WhiteArea M1 (1w)	(size corrected)	Temperature	11.2	3	271	***	80.3	3	353	***	8.0	3	201	***	3.3	3	353	*	119.4	3	393	***
		Sex	25.4	1	271	***	8.4	1	353	**	1.9	1	201	0.17	4.9	1	353	*	7.7	1	393	**
		Temperature x Sex													2.9	3	353	*				

4B: VFW BlackArea M1 (1b)

Effects of developmental temperature on wing pattern element 1b in *ANY*, *SAF*, *MIN*, *PER* and *IBO* (see figure 1 in main text). The mean value is plotted as a function of temperature and error bars represent 95% confidence intervals. Females and males are represented by the solid and dotted lines, respectively.

Minimum adequate models of the effect of developmental temperature and sex on the same trait. Statistical significance is indicated as: * P <0.05, ** P <0.01, *** P <0.001.

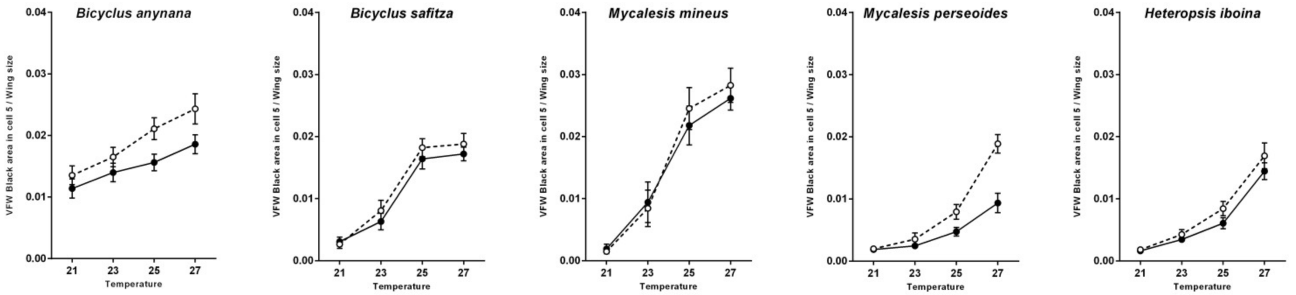

Trait	(transformation)	Fixed effects	F	df	df	P	F	df	df	P	F	df	df	P	F	df	df	P	F	df	df	P
VFW BlackArea M1 (1b)	(size corrected)	Temperature	42.7	3	271	***	287.3	3	353	***	103.7	3	201	***	236.2	3	353	***	224.7	3	393	***
		Sex	44.4	1	271	***	6.0	1	353	*	1.4	1	201	0.24	103.5	1	353	***	16.9	1	393	***
		Temperature x Sex													32.2	3	353	***				

4C: VFW YellowArea M1 (1y)

Effects of developmental temperature on wing pattern element 1y in *ANY*, *SAF*, *MIN*, *PER* and *IBO* (see figure 1 in main text). The mean value is plotted as a function of temperature and error bars represent 95% confidence intervals. Females and males are represented by the solid and dotted lines, respectively.

Minimum adequate models of the effect of developmental temperature and sex on the same trait. Statistical significance is indicated as: * P <0.05, ** P <0.01, *** P <0.001.

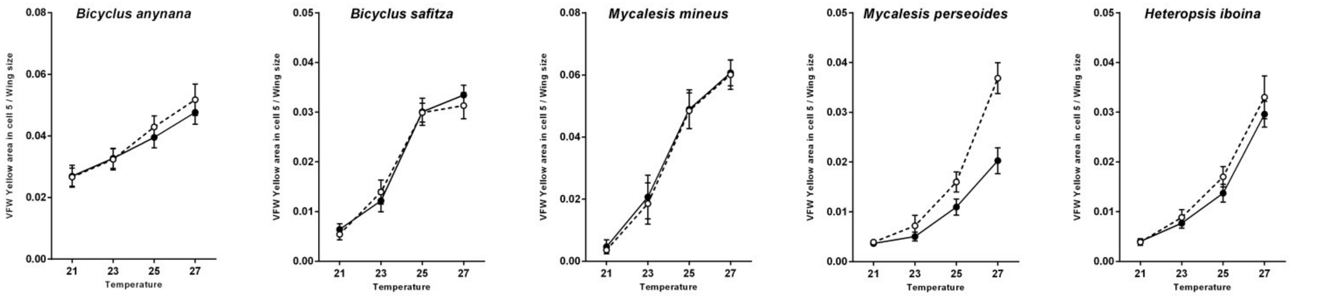

Trait	(transformation)	Fixed effects	F	df	df	P	F	df	df	P	F	df	df	P	F	df	df	P	F	df	df	P
VFW YellowArea M1 (1y)	(size corrected)	Temperature	60.7	3	271	***	344.2	3	353	***	125.9	3	201	***	256.4	3	353	***	213.8	3	393	***
		Sex	1.8	1	271	0.19	0.2	1	353	0.64	0.2	1	201	0.63	82.5	1	353	***	7.9	1	393	**
		Temperature x Sex													25.8	3	353	***				

4D: VFW BlackArea Cu1 (2b)

Effects of developmental temperature on wing pattern element 2b in *ANY*, *SAF*, *MIN*, *PER* and *IBO* (see figure 1 in main text). The mean value is plotted as a function of temperature and error bars represent 95% confidence intervals. Females and males are represented by the solid and dotted lines, respectively.

Minimum adequate models of the effect of developmental temperature and sex on the same trait. Statistical significance is indicated as: * P <0.05, ** P <0.01, *** P <0.001.

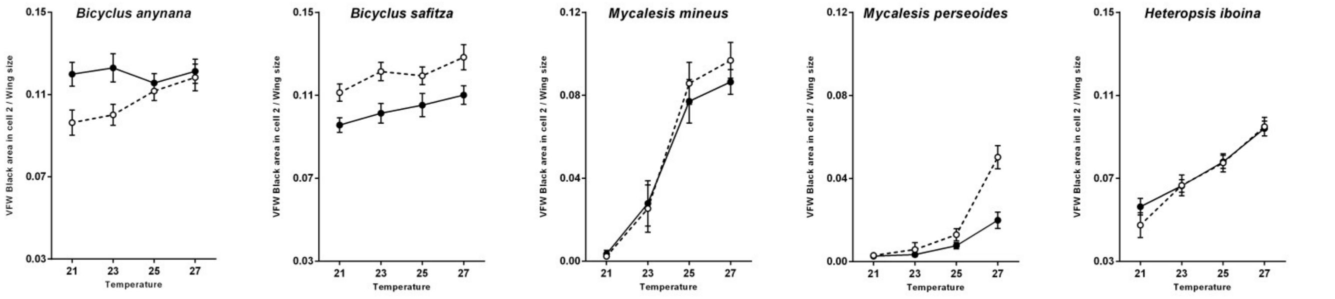

Trait	(transformation)	Fixed effects	F	df	df	P	F	df	df	P	F	df	df	P	F	df	df	P	F	df	df	P
VFW BlackArea Cu1 (2b)	(size corrected)	Temperature	6.0	3	268	***	16.4	3	353	***	121.3	3	201	***	191.1	3	353	***	125.5	3	393	***
		Sex	44.1	1	268	***	112.1	1	353	***	2.3	1	201	0.13	85.0	1	353	***	0.6	1	393	0.43
		Temperature x Sex	8.1	3	268	***									38.4	3	353	***				

4E: VFW Band (3)

Effects of developmental temperature on wing pattern element 3 in *ANY*, *SAF*, *MIN*, *PER* and *IBO* (see figure 1 in main text). The mean value is plotted as a function of temperature and error bars represent 95% confidence intervals. Females and males are represented by the solid and dotted lines, respectively.

Minimum adequate models of the effect of developmental temperature and sex on the same trait. Statistical significance is indicated as: * P <0.05, ** P <0.01, *** P <0.001.

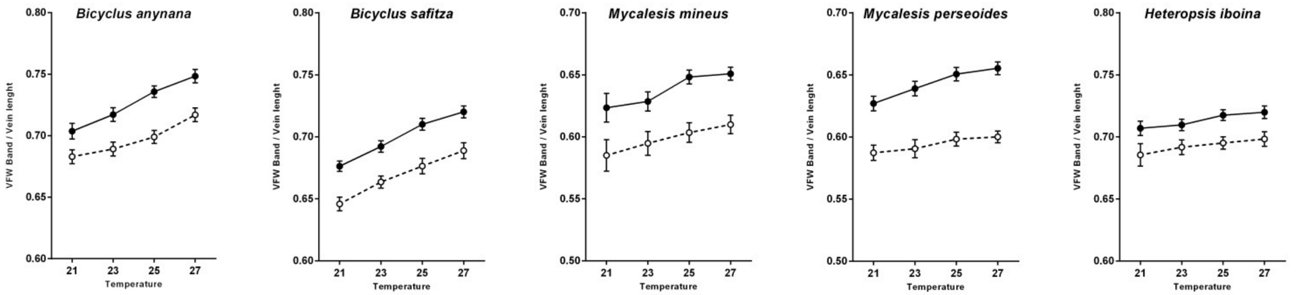

Trait	(transformation)	Fixed effects	F	df	df	P	F	df	df	P	F	df	df	P	F	df	df	P	F	df	df	P
VFW Band (3)	(size corrected)	Temperature	77.6	3	269	***	112.9	3	353	***	21.5	3	198	***	23.1	3	356	***	4.4	3	393	**
		Sex	228.8	1	269	***	292.1	1	353	***	225.7	1	198	***	599.1	1	356	***	113.9	1	393	***
		Temperature x Sex	3.0	3	269	*																

4F: VHW WhiteArea Cu1 (4w)

Effects of developmental temperature on wing pattern element 4w in *ANY*, *SAF*, *MIN*, *PER* and *IBO* (see figure 1 in main text). The mean value is plotted as a function of temperature and error bars represent 95% confidence intervals. Females and males are represented by the solid and dotted lines, respectively.

Minimum adequate models of the effect of developmental temperature and sex on the same trait. Statistical significance is indicated as: * P <0.05, ** P <0.01, *** P <0.001.

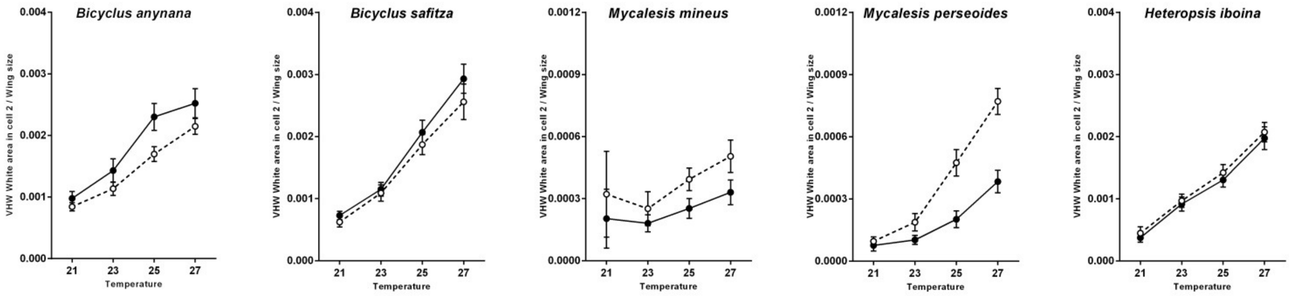

Trait	(transformation)	Fixed effects	F	df	df	P	F	df	df	P	F	df	df	P	F	df	df	P	F	df	df	P
VHW WhiteArea Cu1 (4w)	(size corrected)	Temperature	139.4	3	269	***	273.6	3	353	***	11.6	3	201	***	187.5	3	353	***	177.1	3	393	***
		Sex	40.9	1	269	***	9.4	1	353	**	27.4	1	201	***	153.4	1	353	***	3.3	1	393	0.07
		Temperature x Sex	3.2	3	269	*									24.7	3	353	***				

4G: VHW BlackArea Cu1 (4b)

Effects of developmental temperature on wing pattern element 4b in *ANY*, *SAF*, *MIN*, *PER* and *IBO* (see figure 1 in main text). The mean value is plotted as a function of temperature and error bars represent 95% confidence intervals. Females and males are represented by the solid and dotted lines, respectively.

Minimum adequate models of the effect of developmental temperature and sex on the same trait. Statistical significance is indicated as: * P <0.05, ** P <0.01, *** P <0.001.

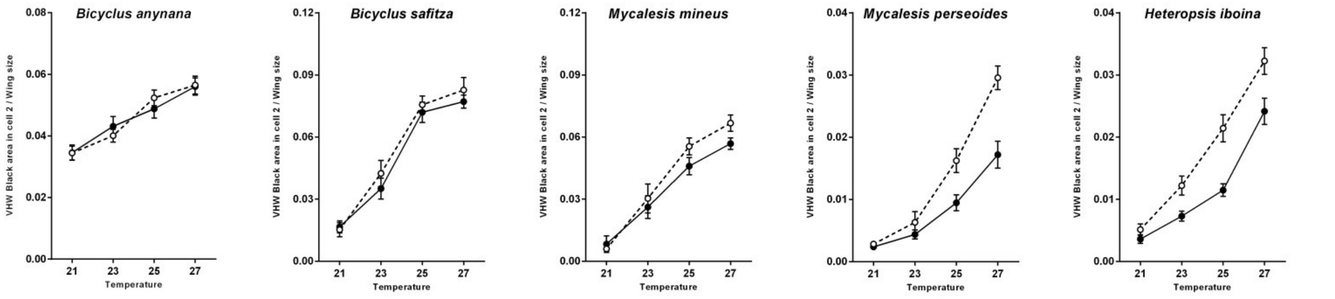

Trait	(transformation)	Fixed effects	F	df	df	P	F	df	df	P	F	df	df	P	F	df	df	P	F	df	df	P
VHW BlackArea Cu1 (4b)	(size corrected)	Temperature	103.9	3	272	***	372.1	3	353	***	158.5	3	201	***	300.8	3	353	***	322.4	3	390	***
		Sex	0.1	1	272	0.82	5.0	1	353	*	17.9	1	201	***	114.0	1	353	***	152.1	1	390	***
		Temperature x Sex													23.7	3	353	***	9.3	3	390	***

4H: VHW YellowArea Cu1 (4y)

Effects of developmental temperature on wing pattern element 4y in *ANY*, *SAF*, *MIN*, *PER* and *IBO* (see figure 1 in main text). The mean value is plotted as a function of temperature and error bars represent 95% confidence intervals. Females and males are represented by the solid and dotted lines, respectively.

Minimum adequate models of the effect of developmental temperature and sex on the same trait. Statistical significance is indicated as: * P <0.05, ** P <0.01, *** P <0.001.

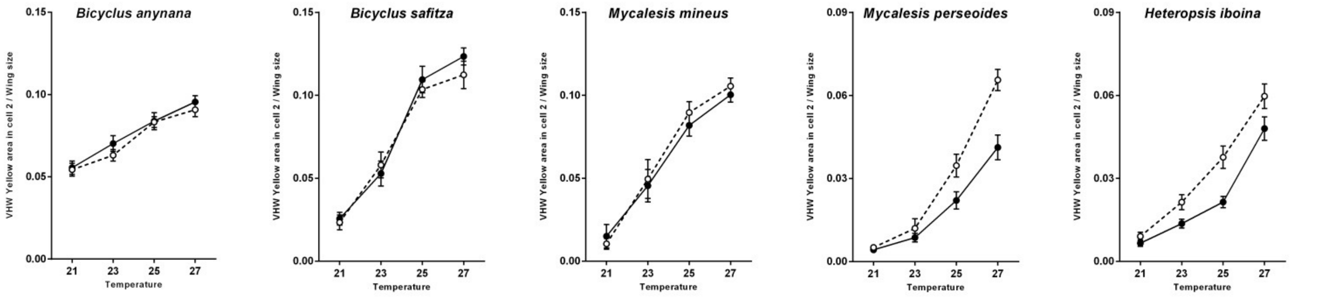

Trait	(transformation)	Fixed effects	F	df	df	P	F	df	df	P	F	df	df	P	F	df	df	P	F	df	df	P
VHW YellowArea Cu1 (4y)	(size corrected)	Temperature	139.3	3	272	***	413.1	3	353	***	172.5	3	201	***	373.8	3	353	***	319.5	3	390	***
		Sex	5.6	1	272	*	2.0	1	353	0.16	3.0	1	201	0.08	93.8	1	353	***	97.8	1	390	***
		Temperature x Sex													20.9	3	353	***	6.3	3	390	***

4I: VHW Band (5)

Effects of developmental temperature on wing pattern element 5 in *ANY*, *SAF*, *MIN*, *PER* and *IBO* (see figure 1 in main text). The mean value is plotted as a function of temperature and error bars represent 95% confidence intervals. Females and males are represented by the solid and dotted lines, respectively.

Minimum adequate models of the effect of developmental temperature and sex on the same trait. Statistical significance is indicated as: * P <0.05, ** P <0.01, *** P <0.001.

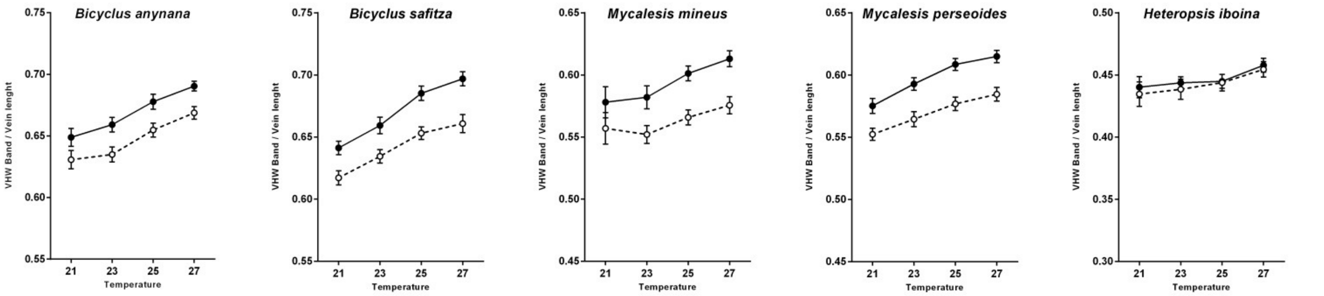

Trait	(transformation)	Fixed effects	F	df	df	P	F	df	df	P	F	df	df	P	F	df	df	P	F	df	df	P
VHW Band (5)	(size corrected)	Temperature	74.8	3	272	***	119.9	3	353	***	29.1	3	198	***	69.0	3	356	***	9.6	3	393	***
		Sex	108.1	1	272	***	188.8	1	353	***	154.7	1	198	***	229.2	1	356	***	2.1	1	393	0.15
		Temperature x Sex																				

4J: DFW WhiteArea M1 (6w)

Effects of developmental temperature on wing pattern element 6w in *ANY*, *SAF*, *MIN*, *PER* and *IBO* (see figure 1 in main text). The mean value is plotted as a function of temperature and error bars represent 95% confidence intervals. Females and males are represented by the solid and dotted lines, respectively.

Minimum adequate models of the effect of developmental temperature and sex on the same trait. Statistical significance is indicated as: * P <0.05, ** P <0.01, *** P <0.001.

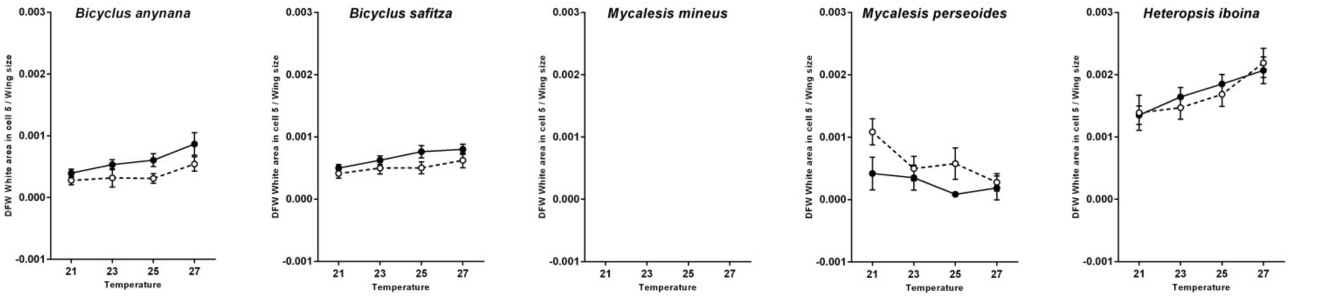

Trait	(transformation)	Fixed effects	F	df	df	P	F	df	df	P	F	df	df	P	F	df	df	P	F	df	df	P
DFW WhiteArea M1 (6w)	(size corrected)	Temperature	17.3	3	242	***	14.6	3	345	***	na	na	na	na	12.0	3	218	***	19.9	3	391	***
		Sex	35.4	1	242	***	27.8	1	345	***	na	na	na	na	24.6	1	218	***	0.9	1	391	0.33
		Temperature x Sex									na	na	na	na	3.7	3	218	*				

4K: DFW BlackArea M1 (6b)

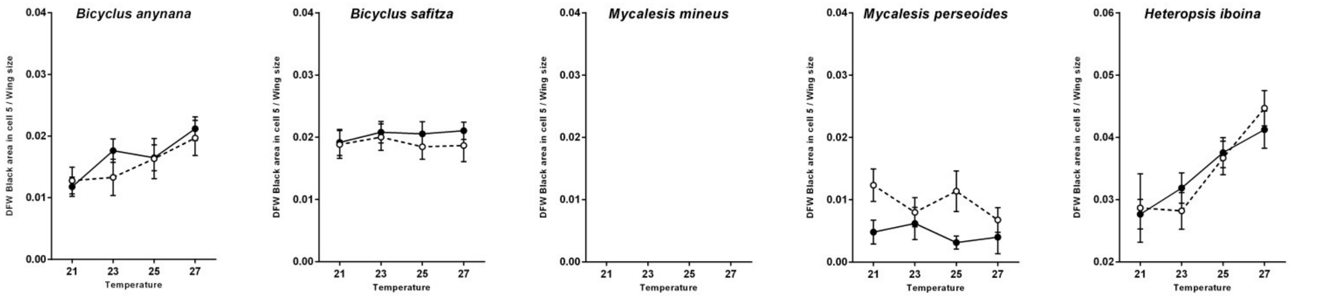

Effects of developmental temperature on wing pattern element 6b in *ANY*, *SAF*, *MIN*, *PER* and *IBO* (see figure 1 in main text). The mean value is plotted as a function of temperature and error bars represent 95% confidence intervals. Females and males are represented by the solid and dotted lines, respectively.

Minimum adequate models of the effect of developmental temperature and sex on the same trait. Statistical significance is indicated as: * P <0.05, ** P <0.01, *** P <0.001.

**Trait**	**(transformation)**	**Fixed effects**	**F**	**df**	**df**	**P**	**F**	**df**	**df**	**P**	**F**	**df**	**df**	**P**	**F**	**df**	**df**	**P**	**F**	**df**	**df**	**P**
DFW BlackArea M1 (6b)	(size corrected)	Temperature	19.1	3	242	***	0.8	3	345	0.47	na	na	na	na	3.0	3	218	*	40.1	3	391	***
		Sex	2.1	1	242	0.15	3.3	1	345	0.07	na	na	na	na	34.1	1	218	***	0.1	1	391	0.75
		Temperature x Sex									na	na	na	na	3.5	3	218	*				

4L: DFW YellowArea M1 (6y)

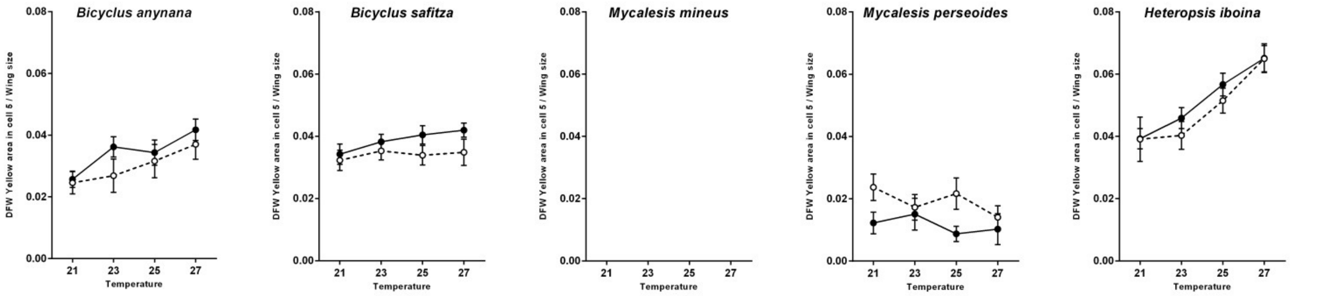

Effects of developmental temperature on wing pattern element 6y in *ANY*, *SAF*, *MIN*, *PER* and *IBO* (see figure 1 in main text). The mean value is plotted as a function of temperature and error bars represent 95% confidence intervals. Females and males are represented by the solid and dotted lines, respectively.

Minimum adequate models of the effect of developmental temperature and sex on the same trait. Statistical significance is indicated as: * P <0.05, ** P <0.01, *** P <0.001.

**Trait**	**(transformation)**	**Fixed effects**	**F**	**df**	**df**	**P**	**F**	**df**	**df**	**P**	**F**	**df**	**df**	**P**	**F**	**df**	**df**	**P**	**F**	**df**	**df**	**P**
DFW YellowArea M1 (6y)	(size corrected)	Temperature	19.2	3	242	***	5.0	3	345	**	na	na	na	na	3.1	3	218	*	54.5	3	391	***
		Sex	9.7	1	242	**	16.5	1	345	***	na	na	na	na	25.3	1	218	***	4.9	1	391	*
		Temperature x Sex									na	na	na	na	3.1	3	218	*				

4M: DFW WhiteArea Cu1 (7w)

Effects of developmental temperature on wing pattern element 7w in *ANY*, *SAF*, *MIN*, *PER* and *IBO* (see figure 1 in main text). The mean value is plotted as a function of temperature and error bars represent 95% confidence intervals. Females and males are represented by the solid and dotted lines, respectively.

Minimum adequate models of the effect of developmental temperature and sex on the same trait. Statistical significance is indicated as: * P <0.05, ** P <0.01, *** P <0.001.

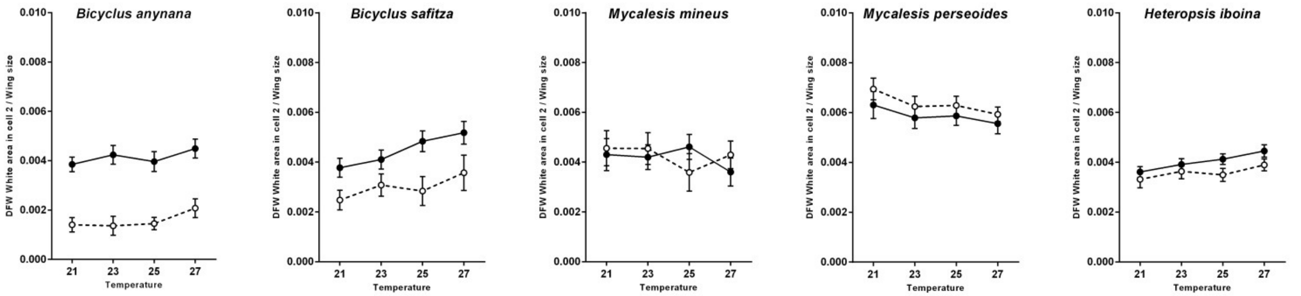

Trait	(transformation)	Fixed effects	F	df	df	P	F	df	df	P	F	df	df	P	F	df	df	P	F	df	df	P
DFW WhiteArea Cu1 (7w)	(size corrected)	Temperature	5.9	3	242	***	11.0	3	345	***	1.2	3	198	0.32	6.8	3	356	***	6.9	3	393	***
		Sex	410.5	1	242	***	78.8	1	345	***	0.0	1	198	0.99	10.7	1	356	**	25.1	1	393	***
		Temperature x Sex									3.7	3	198	*								

4N: DFW BlackArea Cu1 (7b)

Effects of developmental temperature on wing pattern element 7b in *ANY*, *SAF*, *MIN*, *PER* and *IBO* (see figure 1 in main text). The mean value is plotted as a function of temperature and error bars represent 95% confidence intervals. Females and males are represented by the solid and dotted lines, respectively.

Minimum adequate models of the effect of developmental temperature and sex on the same trait. Statistical significance is indicated as: * P <0.05, ** P <0.01, *** P <0.001.

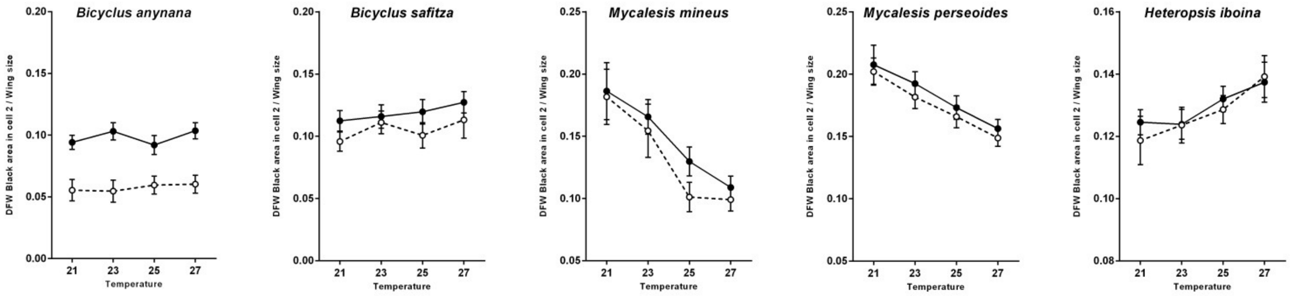

Trait	(transformation)	Fixed effects	F	df	df	P	F	df	df	P	F	df	df	P	F	df	df	P	F	df	df	P
DFW BlackArea Cu1 (7b)	(size corrected)	Temperature	2.5	3	242	0.06	4.1	3	345	**	45.9	3	201	***	44.8	3	356	***	13.1	3	393	***
		Sex	246.4	1	242	***	15.8	1	345	***	10.3	1	201	**	5.5	1	356	*	0.6	1	393	0.42
		Temperature x Sex																				

4O: DFW YellowArea Cu1 (7y)

Effects of developmental temperature on wing pattern element 7y in *ANY*, *SAF*, *MIN*, *PER* and *IBO* (see figure 1 in main text). The mean value is plotted as a function of temperature and error bars represent 95% confidence intervals. Females and males are represented by the solid and dotted lines, respectively.

Minimum adequate models of the effect of developmental temperature and sex on the same trait. Statistical significance is indicated as: * P <0.05, ** P <0.01, *** P <0.001.

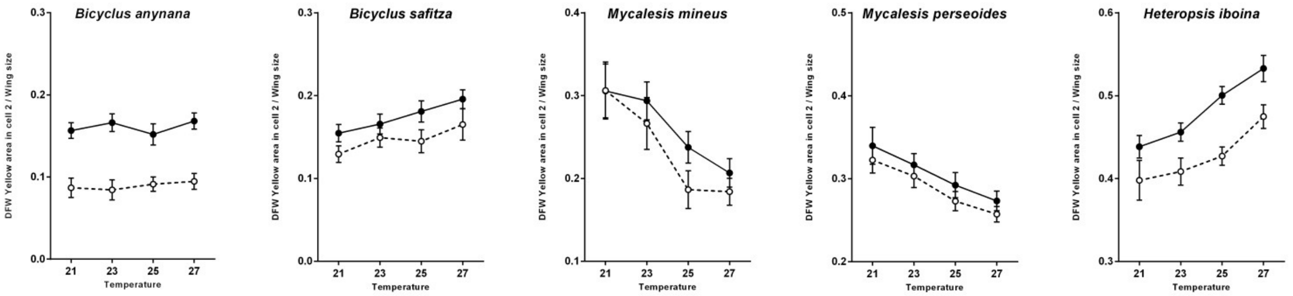

Trait	(transformation)	Fixed effects	F	df	df	P	F	df	df	P	F	df	df	P	F	df	df	P	F	df	df	P
DFW YellowArea Cu1 (7y)	(size corrected)	Temperature	2.0	3	242	0.11	14.0	3	345	***	34.5	3	201	***	34.3	3	356	***	46.8	3	393	***
		Sex	345.3	1	242	***	36.8	1	345	***	14.3	1	201	***	11.6	1	356	***	134.8	1	393	***
		Temperature x Sex																				

4P: PC1 ventral wing elements

Effects of developmental temperature on PC1 (ventral wing pattern elements) in *ANY*, *SAF*, *MIN*, *PER* and *IBO* (see figure 1 in main text). The mean value is plotted as a function of temperature and error bars represent 95% confidence intervals. Females and males are represented by the solid and dotted lines, respectively.

Minimum adequate models of the effect of developmental temperature and sex on the same trait. Statistical significance is indicated as: * P <0.05, ** P <0.01, *** P <0.001.

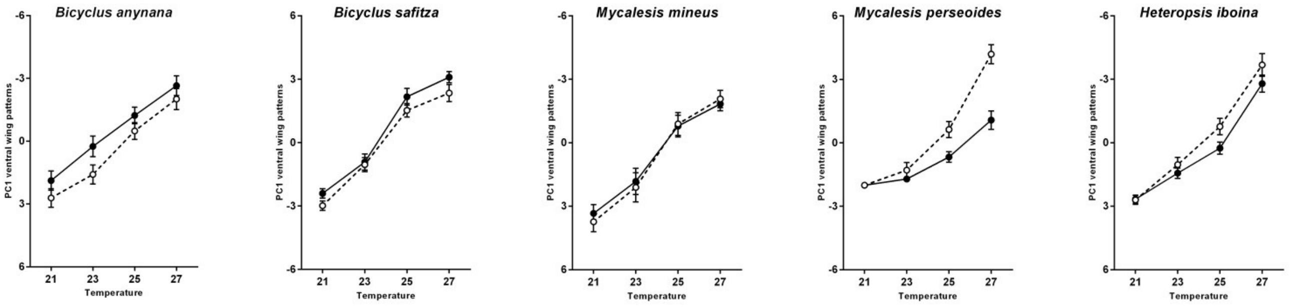

Trait	(transformation)	Fixed effects	F	df	df	P	F	df	df	P	F	df	df	P	F	df	df	P	F	df	df	P
PC1 ventral wing elements	(size corrected)	Temperature	166.42	3	271	***	521.2	3	353	***	149.4	3	200	***	339.4	3	353	***	364.1	3	390	***
		Sex	32.35	1	271	***	21.2	1	353	***	0.0	1	200	0.95	128.7	1	353	***	27.4	1	390	***
		Temperature x Sex													34.9	3	353	***	3.0	3	390	*
