# Supplementary material for: Conserved patterns of integrated developmental plasticity in a group of polyphenic tropical butterflies
Source: BMC Evol Biol. 2017 Feb 27;17:59. doi: 10.1186/s12862-017-0907-1 (PMC5327525; doi:10.1186/s12862-017-0907-1)
Supplement: Additional file 5: — Summary table of the plastic responses to developmental temperature in mycalesine butterflies. (PDF 29 kb) [file 12862_2017_907_MOESM5_ESM.pdf]

Additional file 5.

Summary table of the plastic responses to developmental temperature in mycalesine butterflies. Traits that make up the proposed functional suite (FS) are indicated in bold. The direction of the response to the temperature gradient is represented by blue shading when the relationship between the traits and developmental temperature is negative (-), red shading signifies traits which are positively correlated to developmental temperature (+). Traits which did not respond to developmental temperature are indicated with an 'x'. The right-hand column refers to works in which the role of ecdysteroids in mediating plastic responses is studied in *B. anynana*.

FS	Traits	*IBO*	*ANY*	*SAF*	*MIN*	*PER*	Ecdysone
+	**Development time** [A]	−	−	−	−	−	[45, 49]
+	**Body size** [B]	−	−	− [F]	−	−	[47]
+	**Abdomen ratio**	+	+	+	+	+	[49]
+	**Ventral forewing** [C]	+	+	+	+	+	[46-48]
+	**Ventral hindwing** [D]	+	+	+	+	+	[46-48]
−	Fat content	x	−	x	x	−	[49]
−	Dorsal hindwing [E]	+	x	x	−	−	[47]

[A] Larval development time (LDT), pupal development time (PDT) and total development time (DT). [B] Pupal weight (PW), total wing area (WA) and dry mass (DM). [C] Ventral forewing eyespots (1,2) and band (3), numbered as in figure 1 of the main text. [D] Ventral hindwing eyespot (4) and band (5), numbered as in figure 1 of the main text. [E] Dorsal hindwing eyespot (6), numbered as in figure 1 of the main text. [F] Dry mass (DM) was not significantly affected by developmental temperature in *B. safitza*.
